# RNA G-quadruplex formed in SARS-CoV-2 used for COVID-19 treatment in animal models

**DOI:** 10.1038/s41421-022-00450-x

**Published:** 2022-09-06

**Authors:** Geng Qin, Chuanqi Zhao, Yan Liu, Cheng Zhang, Guang Yang, Jie Yang, Zhao Wang, Chunyu Wang, Changchun Tu, Zhendong Guo, Jinsong Ren, Xiaogang Qu

**Affiliations:** 1 Laboratory of Chemical Biology and State Key Laboratory of Rare Earth Resource Utilization, Changchun Institute of Applied Chemistry, Chinese Academy of Sciences, Changchun, Jilin China; 2 University of Science and Technology of China, Hefei, Anhui China; 3 Changchun Veterinary Research Institute, Chinese Academy of Agricultural Sciences, Changchun, Jilin China; 4 Hebei Agricultural University, College of Veterinary Medicine, 2596 Lucky South Street, Baoding, Hebei China; 5 State Key Laboratory of Supramolecular Structure and Materials, Jilin University, Changchun, Jilin China

**Keywords:** RNA folding, Mechanisms of disease

## Abstract

The ongoing COVID-19 pandemic has continued to affect millions of lives worldwide, leading to the urgent need for novel therapeutic strategies. G-quadruplexes (G4s) have been demonstrated to regulate life cycle of multiple viruses. Here, we identify several highly conservative and stable G4s in SARS-CoV-2 and clarify their dual-function of inhibition of the viral replication and translation processes. Furthermore, the cationic porphyrin compound 5,10,15,20-tetrakis-(N-methyl-4-pyridyl)porphine (TMPyP4) targeting SARS-CoV-2 G4s shows excellent antiviral activity, while its N-methyl-2-pyridyl positional isomer TMPyP2 with low affinity for G4 has no effects on SARS-CoV-2 infection, suggesting that the antiviral activity of TMPyP4 attributes to targeting SARS-CoV-2 G4s. In the Syrian hamster and transgenic mouse models of SARS-CoV-2 infection, administration of TMPyP4 at nontoxic doses significantly suppresses SARS-CoV-2 infection, resulting in reduced viral loads and lung lesions. Worth to note, the anti-COVID-19 activity of TMPyP4 is more potent than remdesivir evidenced by both in vitro and in vivo studies. Our findings highlight SARS-CoV-2 G4s as a novel druggable target and the compelling potential of TMPyP4 for COVID-19 therapy. Different from the existing anti-SARS-CoV-2 therapeutic strategies, our work provides another alternative therapeutic tactic for SARS-CoV-2 infection focusing on targeting the secondary structures within SARS-CoV-2 genome, and would open a new avenue for design and synthesis of drug candidates with high selectivity toward the new targets.

## Introduction

The ongoing pandemic of coronavirus disease 2019 (COVID-19), caused by a novel severe acute respiratory syndrome coronavirus 2 (SARS-CoV-2)^1,2^, has resulted in more than 570 million infections and 6 million deaths worldwide by the end of July 2022 in 223 countries. Despite intensive countermeasures implemented around the world, the morbidity and mortality of COVID-19 remain high, and the emergence and rapid spread of various SARS-CoV-2 variants that are more contagious has triggered new waves in many countries.

Despite implementing large-scale vaccination efforts around the world, the SARS-CoV-2 variants have reduced the efficacy of vaccines, posing serious challenges to the control of the COVID-19 pandemic^3^. Thus, developing safe and effective antiviral drugs is still a high priority. So far, the development of anti-SARS-CoV-2 agents has been mainly focused on RNA-dependent RNA polymerase, protease, viral structure proteins and several host proteins^4–8^. Remdesivir has at the earliest been reported to exhibit benefits for patient outcomes in clinical trials^9^, but it showed relatively low efficacy in further large-scale studies^10^. In fact, the therapy by using single drug targeting SARS-CoV-2 tends to be suboptimal. Paxlovid (PF-07321332 + ritonavir), which is a ritonavir-boosted protease inhibitor, would reduce the risk of hospitalization or death in 90% of patients with mild to moderate disease at the conclusion of its EPIC study (Phase 2/3)^11^. Given that frequent emergence of various SARS-CoV-2 variants would limit the efficacy of vaccines and drugs, the development of more antiviral strategies and drugs for COVID-19 is still urgent needed, as is the establishment of combinatorial regimens using different classes of agents that target multiple steps in the life cycle of SARS-CoV-2 to improve efficacy and reduce the potential for the emergence of drug-resistant variants.

G-quadruplexes (G4s) are non-canonical nucleic acid secondary structures that can form within DNA or RNA guanine (G)-rich strands^12^. G4s have been found in the genome of humans, yeasts, bacteria and viruses etc.^13^, and involve in regulating various biological processes^12,14^. Recently, G4s have been identified as potential antiviral targets of multiple viruses, such as Hepatitis C virus (HCV), Zika virus (ZIKV) and Ebola virus (EBOV)^15^. Several studies have predicted many putative G4-forming sequences (PQSs) in SARS-CoV-2 genome^16–20^. Whether G4 can form in the context of SARS-CoV-2 infection and the formed G4 can act as the therapeutic targets of COVID-19 has become the focus, leading to much attention in this field.

In this study, we identified several highly conservative and stable G4s in SARS-CoV-2, confirmed the formation of RNA G4s in the context of SARS-CoV-2 infection and clarified their dual-function of inhibiting viral replication and translation processes. Furthermore, the inhibitory effects of SARS-CoV-2 G4s can be enhanced by G4-specific ligands 5,10,15,20-tetrakis-(Nmethyl-4-pyridyl)porphine (TMPyP4). TMPyP4 has shown effective antiviral activity in Vero E6 cells, syrian hamster and human angiotensin-converting enzyme 2 (hACE2) transgenic mouse model of SARS-CoV-2 infection with no observable toxicity. The antiviral activity of TMPyP4 is more potent than remdesivir both in vitro and in vivo. Different from the existing anti-SARS-CoV-2 therapeutic strategies, our finding provides another alternative therapeutic strategy for SARS-CoV-2 infection focusing on targeting functional secondary structures within SARS-CoV-2 genome, besides targeting enzymes or proteins.

## Results

### Identification and annotation of G4s in SARS-CoV-2 whole genome

**Fig. 1:**
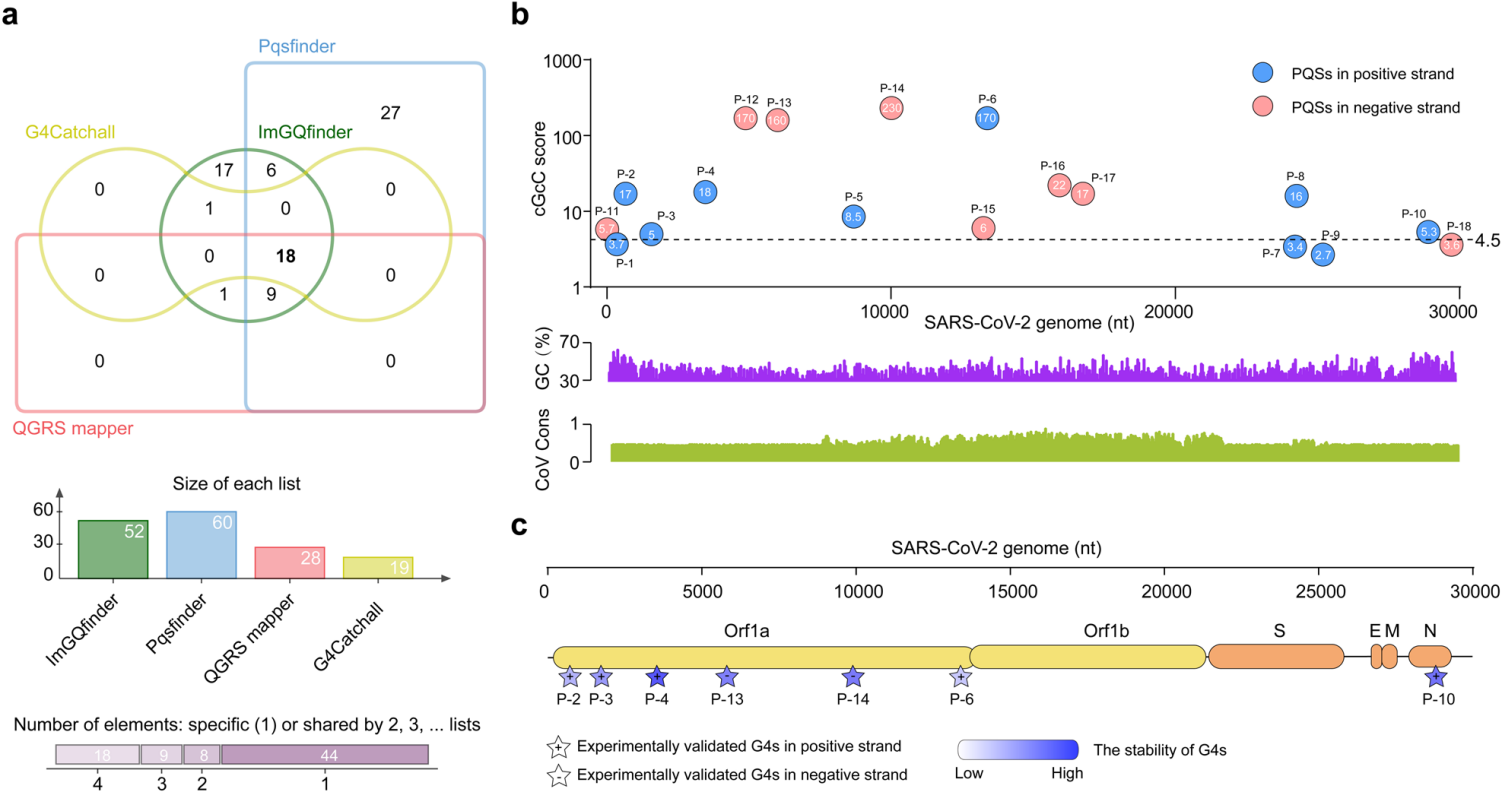
Detection and annotation of G-quadruplexes (G4s) in SARS-CoV-2 genome. **a** Top, edwards’ Venn diagram showing the overlap of the putative G4-forming sequences (PQSs) predicted by four independent softwares (G4catchall, QGRS mapper, Pqsfinder and ImGQfinder) in the genome of SARS-CoV-2. Bottom, charts showing the list size and intersection size repartition. **b** The location and score of the SARS-CoV-2 PQSs. Top, the cGcC score and location of SARS-CoV-2 PQSs. The cG/cC score of PQSs is calculated by using G4RNA screener. Bottom, the GC percent track and multiple alignments of 7 human coronavirus sequences. cGcC, consecutive G over consecutive C ratio. **c** The location and stability of experimentally validated G4s in the genome of SARS-CoV-2. The stability of G4s is detected by thermal melting assays.

In an effort to investigate whether G4s can be promising therapeutic targets for COVID-19, we determined to screen all G4s of SARS-CoV-2 genome by an integrative strategy (Supplementary Fig. S1a). We first predicted the PQSs within viral genome of SARS-CoV-2 by combing four independent G4 prediction softwares (Fig. 1a), and then evaluated the G4 folding capabilities by the consecutive G over consecutive C ratio (cGcC), G4Hunter (G4H) and G4 neural network (G4NN) scores as described in Materials and Methods. Seven PQSs in positive-sense strands and 7 PQSs in negative-sense strands were identified, which are evidenced by the observation that at least one of the cGcC, G4H and G4NN scores is above the threshold (Fig. 1b; Supplementary Fig. S1b, c and Tables S1 and S2). By using N-methyl mesophorphyrin IX (NMM) (Supplementary Fig. S2a), a well-known fluorescent G4-specific ligand, we next screened the stable G4s in vitro among the 14 PQSs. Seven out of the 14 PQSs can enhance the fluorescence of NMM in K^+^ buffer (Supplementary Fig. S2b), suggesting the formation of G4s. Furthermore, circular dichroism (CD) spectroscopy showed that the 7 PQSs form a parallel G4s with a negative CD peak around 240 nm and a positive peak around 263 nm (Supplementary Fig. S3a); commonly-used thermal melting assays indicated that the melting temperature (*T*_m_) of the 7 PQSs range from 41 °C to 63 °C (Supplementary Fig. S3b), suggesting their survival ability under physiological conditions.

Given that the accumulated mutations of SARS-CoV-2 may result in the resistance for various drugs and vaccines^21,22^, we next determined the conservation of the 7 PQS sites across strains by retrieving 3969 complete SARS-CoV-2 genomic sequences (Supplementary Fig. S4a). We observed that all the PQSs are well maintained through the SARS-CoV-2 strains (Supplementary Fig. S4b). In addition, we also resorted to aligning 7 known human coronavirus genomic sequences for identification of conserved PQSs throughout the family and found that the PQS candidates show moderate or low conservation throughout the human coronavirus (Supplementary Fig. S4c). However, a lot of PQSs were identified in the 7 human coronavirus genomes by our integrative strategy (Supplementary Figs. S5a–l and S6), suggesting that G4s commonly exist in coronavirus genome and can be identified as the potential targets for the development of drugs against coronavirus. In the following studies, we chose P4, which is the most stable among the 7 PQSs, as a representative to investigate the behaviors of G4s in detail.

### Identification of SARS-CoV-2 genomic G4 formation

**Fig. 2:**
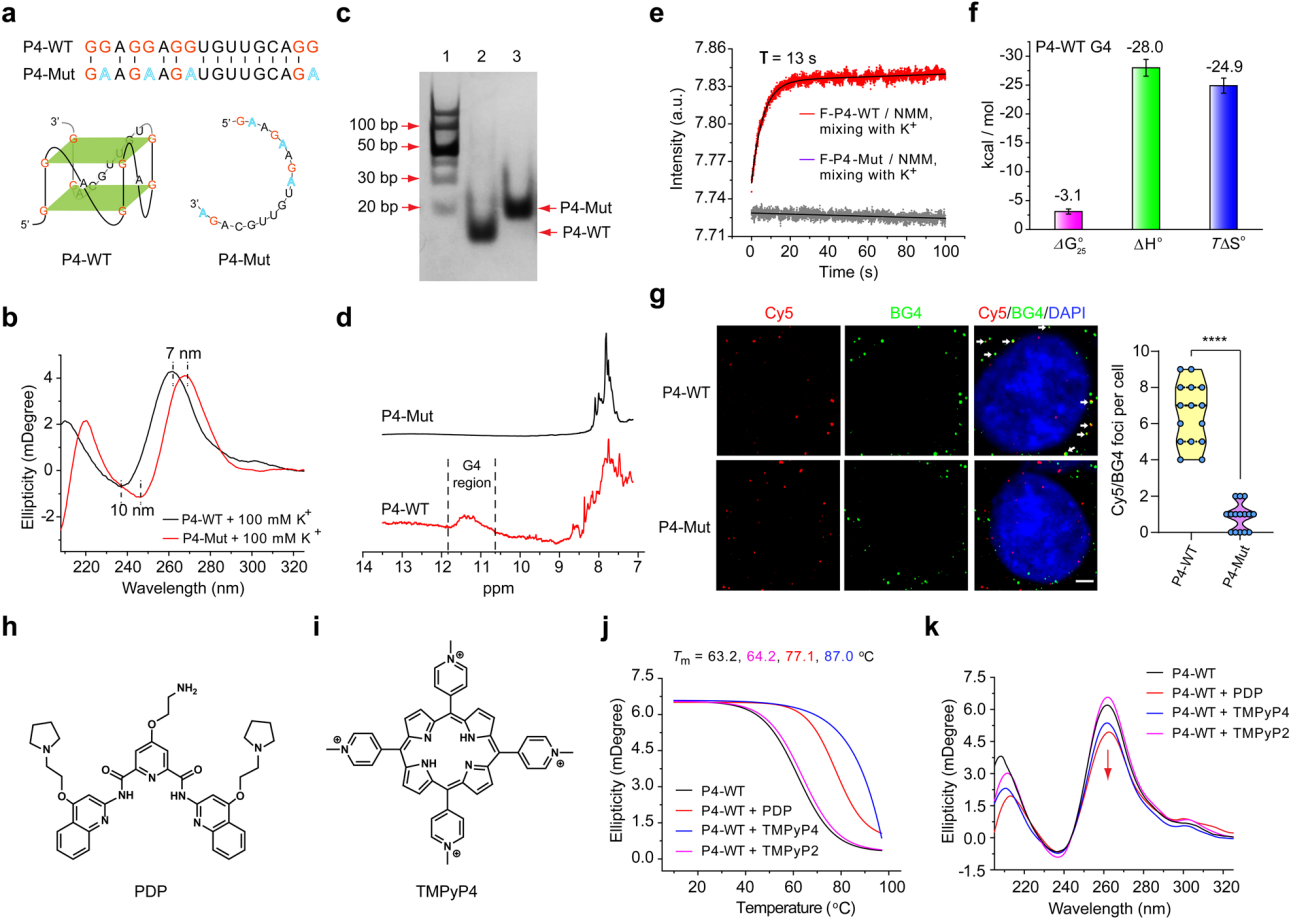
Characterization of P4 G4 formation. **a** Top, the sequences of P4 wild-type (P4-WT) and P4 mutant (P4-Mut). Bottom, the schematic representation of the proposed P4-WT and P4-Mut structures. **b** CD spectroscopy of P4-WT and P4-Mut. CD, circular dichroism. **c** The formation of P4 G4 detected by nondenaturing polyacrylamide gel electrophoresis experiments. Lane 1, DNA ladder; Lane 2, P4-WT; Lane 3, P4-Mut. **d** The formation of P4 G4 detected by ^1^H NMR. NMR, nuclear magnetic resonance. **e** The kinetic folding process of P4 G4 detected by the stopped-flow assays. **f** The thermodynamic parameters of P4 G4 formation estimated from the melting measurements. **g** The formation of P4 G4 in live cells detected by immunofluorescence assays. Left, the white arrows indicated the colocalized foci of Cy5-labeled RNA (red) with BG4 (green). Scale bars, 4 μm. Right, Cy5/BG4 foci number was quantified. ****P < 0.0001. **h**, **i** Chemical structures of PDP (**h**) and TMPyP4 (**i**). **j** CD thermal melting curves of P4-WT (1.5 μM) with PDP (1.5 μM), TMPyP4 (1.5 μM) or TMPyP2 (1.5 μM). **k** CD spectroscopy of P4-WT (1.0 μM) without or with PDP (1.0 μM), TMPyP4 (1.0 μM) or TMPyP2 (1.0 μM).

We further adopted several methods to verify G4 formation of P4. We first synthesized P4 wild-type RNA (P4-WT) and P4 mutant RNA (P4-Mut, with G/A mutations) (Fig. 2a). CD measurements showed that the P4-WT could form parallel G4s, but not for P4-Mut (Fig. 2b). Furthermore, we found that P4-WT migrated faster than P4-Mut (Fig. 2c), suggesting that P4-WT folds into a compact RNA secondary structure. The *T*m of P4-WT did not change with increasing RNA concentrations, indicating that P4 G4 is intramolecularly formed (Supplementary Fig. S7a). The ^1^H nuclear magnetic resonance (NMR) spectra of P4-WT, but not P4-Mut, showed broad imino peaks between 10.5 and 12 ppm, indicating the G4 formation of P4-WT (Fig. 2d). The stopped-flow assays showed that P4 G4 can form within 13 s, following a moderate folding kinetics (Fig. 2e). And the thermodynamic parameters of P4 G4 formation indicated that the formation of G4 was driven by enthalpy-entropy compensation (Fig. 2f; Supplementary Fig. S7b). Finally, we studied the formation of P4 G4 in live cells. RNA G4s were visualized by a well-known G4-specific antibody BG4 with DNase treatment that eliminated the DNA. We observed co-localization between Cy5-labeled P4-WT and BG4 fluorescence, but not that between Cy5-labeled P4-Mut and BG4 fluorescence (Fig. 2g). Taken together, these results demonstrated that P4 could form stable unimolecular G4s.

### G4-specific ligands bind and stabilize SARS-CoV-2 G4

Along with the constantly illustrating of G4s biological functions, the development of G4-specific ligands has also been intensified^15^. G4-specific ligands, e.g., pyridostatin derivative (PDP) and TMPyP4, have been used to target G4s in the genome of various viruses (Fig. 2h, i). We next evaluated the interaction of these G4-specific ligands with SARS-CoV-2 G4s. We found that both PDP and TMPyP4 significantly enhanced the thermal stability of P4 G4 (Fig. 2j), demonstrating the strong interactions between P4 G4 and PDP or TMPyP4. TMPyP2, which is a positional isomer of TMPyP4 with low affinity for G4 DNA (Supplementary Fig. S8), showed no effects on the thermal stability of P4 G4 (Fig. 2j). In addition, the CD peak position of P4 G4 was unchanged in the presence of PDP, TMPyP4 or TMPyP2 (Fig. 2k), implying that PDP, TMPyP4 or TMPyP2 binding did not destroy the parallel structure of P4 G4. Taken together, these results indicated that PDP and TMPyP4 could bind and stabilize P4 G4s.

### G4-specific ligands inhibit viral RNA translation and replication by targeting G4s

The verification of P4 G4 formation in SARS-CoV-2 genome promoted us to further examine the function of G4s on SARS-CoV-2 virus life cycle. P4 locates in the open reading frames (ORFs) of SARS-CoV-2 replicase gene. After attachment and entry into host cells, the first step for the virus is the translation of the replicase gene from the virion genomic RNA^23^. Given that G4s in the ORFs contribute to translation-related processes, such as elongation, ribosomal frameshift and no-go mRNA decay etc.^24^, we speculated that P4 G4 locating in ORFs of replicase gene may inhibit the translation of replicase gene and the polyproteins expression.

**Fig. 3:**
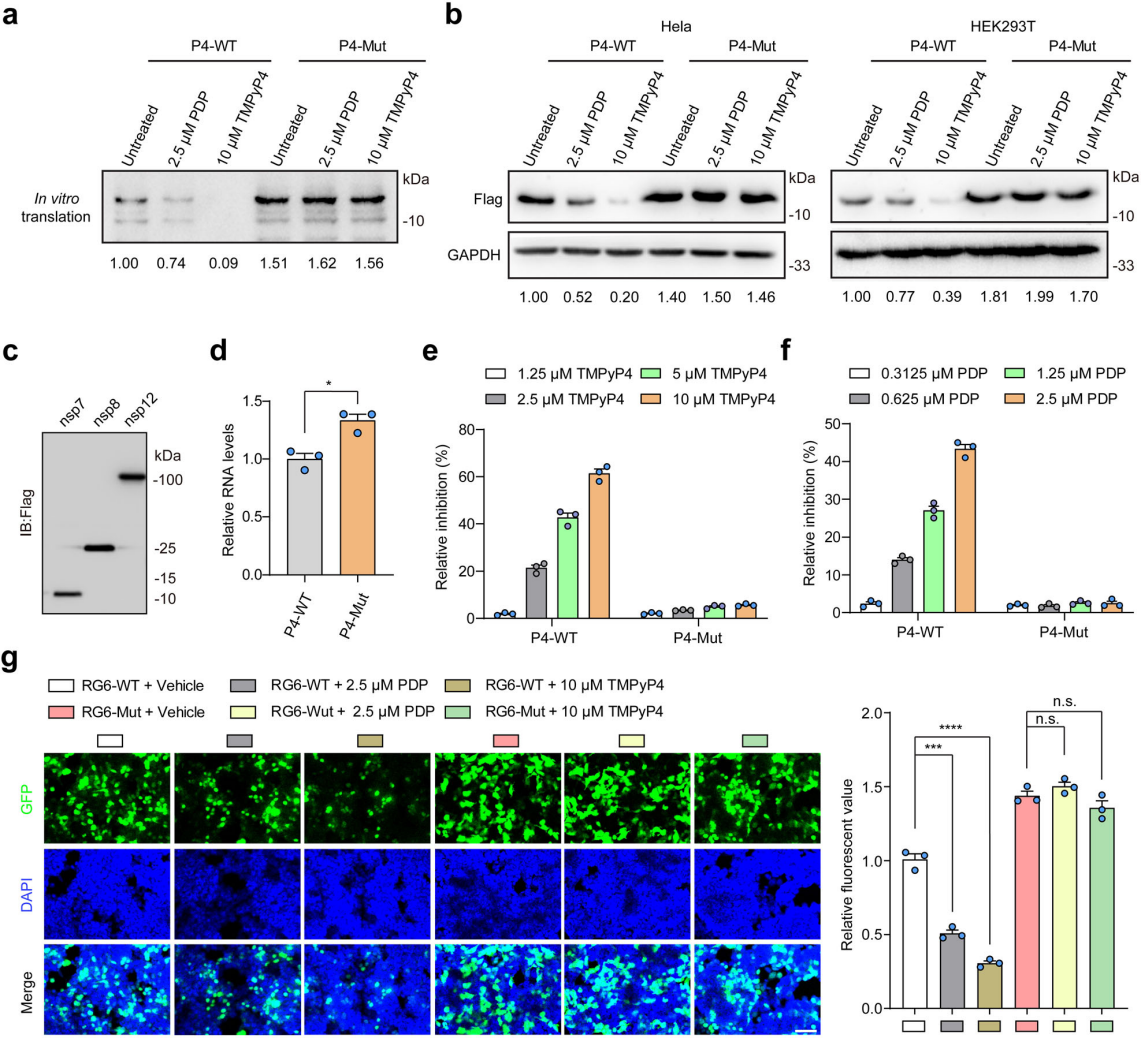
Formation of P4 G4 inhibits SARS-CoV-2 RNA translation and replication processes. **a** TMPyP4 or PDP treatment inhibits the translation of mRNA with P4 G4 by performing IVT assays. **b** TMPyP4 or PDP treatment inhibits the protein expression by targeting P4 G4 in HEK293T and Hela cells. **c** The protein expression of nsp7, nsp8 and nsp12, detected by western blotting assays. **d** The formation of P4 G4 inhibits the replication of RNA with P4 G4 in HEK293T cells, detected by qRT-PCR assays. **e**, **f** TMPyP4 (**e**) or PDP (**f**) treatment inhibits the replication of RNA with P4 G4 in HEK293T cells in a dose-dependent manner, detected by qRT-PCR assays. **g** HEK293T cells infected with P4-EGFP lentivirus were treated with or without 10 μM TMPyP4 or 2.5 μM PDP. Left, representative confocal images. Right, the relative fluorescent value. Scale bars, 100 μm. Data are shown as means ± SEM of three independent experiments, two-tailed Student’s *t*-test. n.s. not significant. ^*^P < 0.05, ^***^P < 0.001, ^****^P < 0.0001.

To this end, the in vitro translation (IVT) assays were performed (Supplementary Fig. S9a). The SARS-CoV-2 genome sequences with P4 G4 or mutant P4 G4 sites were cloned into pGEM-T esay vector and used for IVT assays. We found that the translational efficiency of P4-WT was lower than that of P4-Mut (Fig. 3a; Supplementary Fig. S9b). Meanwhile, both PDP and TMPyP4 treatment inhibited the IVT of P4-WT, but not for P4-Mut (Fig. 3a). Furthermore, the same sequences with P4-WT or P4-Mut were cloned into pCAG-Flag vector to investigate the effects of P4 G4 on the protein expression in living cells. Consistent with the results of IVT assays, the protein expression of P4-WT was less than that of P4-Mut, and PDP or TMPyP4 decreased the protein levels of P4-WT in HeLa and HEK293T cells in a concentration-dependent manner (Fig. 3b; Supplementary Fig. S9c–e).

Following the translation and assembly of the viral replicase complexes, SARS-CoV-2 negative-strand intermediates were synthesized by positive-strand RNA to produce genomic and sub-genomic RNAs^23^. It had been reported that RNA-dependent RNA polymerase (RdRp) can be inhibited by G4s during the elongation phase of RNA synthesis due to steric hindrance^25^. Thus, we speculated that P4 G4 formation may inhibit viral RNA replication. To this end, we performed RNA replication assays by using SARS-CoV-2 RdRp (nsp12, nsp7 and nsp8) (Fig. 3c; Supplementary Fig. S10a). We found that the RNA levels of P4-WT were lower than that of P4-Mut (Fig. 3d). In addition, both PDP and TMPyP4 could significantly inhibit the replication of P4-WT compared with P4-Mut in a dose-dependent manner (Fig. 3e, f).

We further conducted a single-round lentiviral vector with P4-WT or P4-Mut fused to the N terminus of an EGFP reporter gene. After viral packaging, virus supernatant was used to infect HEK293 cells, and confocal fluorescence microscopy study was performed. We observed that PDP or TMPyP4 treatment inhibited the P4-WT EGFP fluorescence intensity, but not P4-Mut (Fig. 3g). Meanwhile, TMPyP2 treatment showed no effects on protein expression, RNA replication or EGFP fluorescence of P4-WT (Supplementary Fig. S10b–d). Collectively, these results suggested that P4 G4 formation may inhibit the viral replication and translation processes.

As a representative, we confirmed the roles of P4 G4 in SARS-CoV-2 life cycle. We also identified other three G4 sites (P2, P3 and P6) in replicase gene, one G4 site (P10) in nucleocapsid phosphoprotein (N) and two G4 sites (P13 and P14) in SARS-CoV-2 negative-sense strand. We next investigated whether the other G4s are involved in regulating SARS-CoV-2 life cycle. Indeed, TMPyP4 treatment inhibited EGFP expression by targeting P2, P3 and P6 G4s, but not P1 (Supplementary Fig. S11a–e). Due to the roles of negative-strand intermediates in the production of genomic and sub-genomic RNAs, we confirmed that TMPyP4 treatment suppressed RNA replication by targeting p13 and p14 G4s (Supplementary Fig. S11f–h). These results indicate that the other identified G4s may also participate in modulating the life cycle of SARS-CoV-2 and the synergistic effects of multi-G4s may enhance the antiviral activity of G4-specific ligands.

### Antiviral results of G4-specific ligands in vitro

**Fig. 4:**
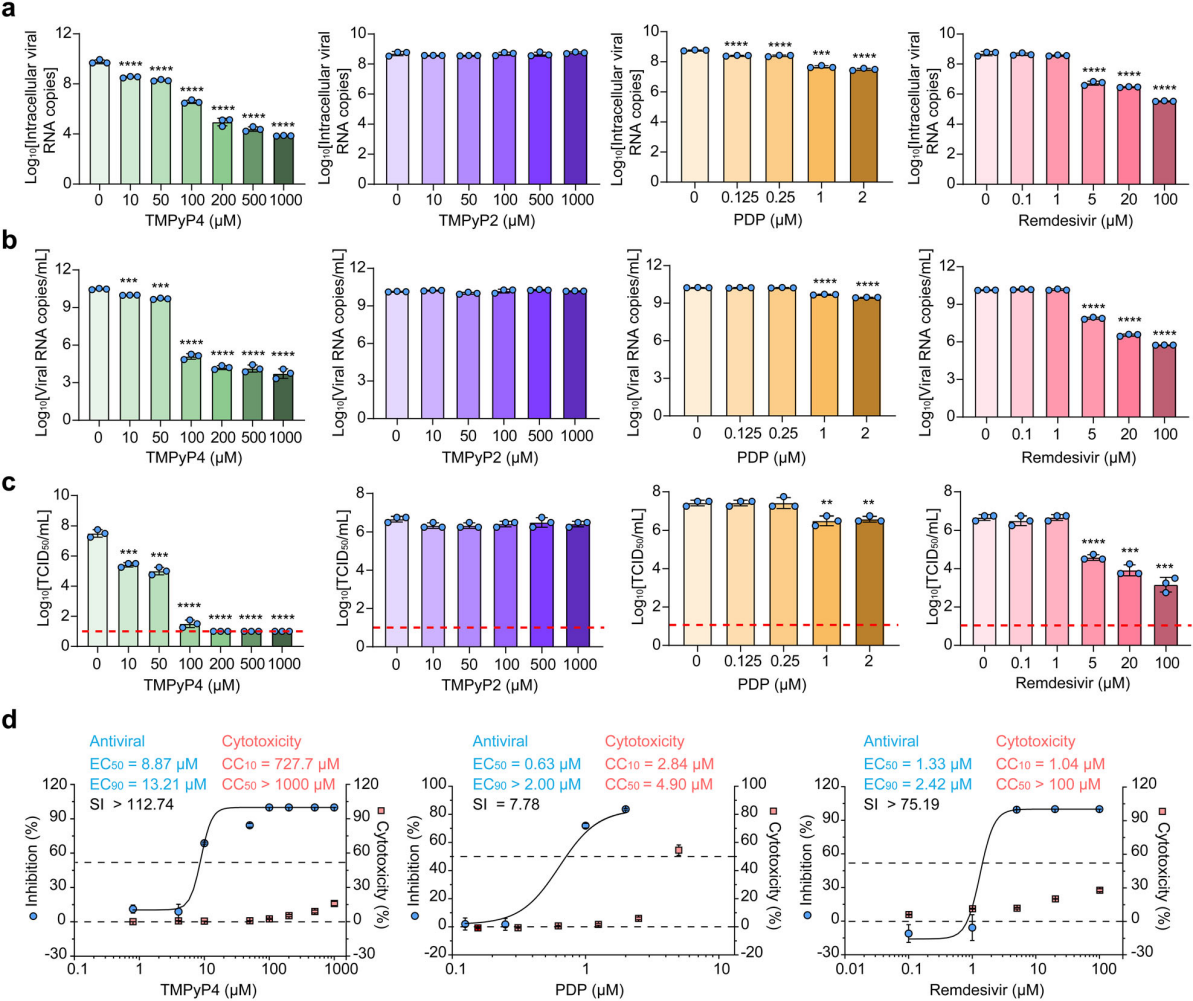
Anti-SARS-CoV-2 activity of TMPyP4 in cell-based assays. **a**–**c** Vero E6 cells infected with SARS-CoV-2 (10^3^ TCID_50_ virus/mL) were pretreated with different concentrations of TMPyP4, TMPyP2, PDP or remdesivir. At 72 h post infection, viral RNA copies in cell lysates (**a**) and in cell culture supernatants (**b**) were detected by qRT-PCR. Viral titers (log_10_TCID_50_/mL) (**c**) were quantified by TCID_50_. Lower limit of detection for viral titers is indicated with a red dotted line. TCID_50_, median tissue culture infectious dose. **d** Fitting viral loads (viral RNA copies/mL) and cell viability under increasing concentration of TMPyP4 to determine the EC_50_ and CC_50_ of TMPyP4, PDP and remdesivir. The drugs cytotoxicity was detected by SRB assays. EC_50_, half-maximal effective doses. EC_90_, 90% maximal effective doses. CC_10_, 10% cytotoxic concentration. CC_50_, half-cytotoxic concentration. Data are shown as means ± SEM of three independent experiments, two-tailed Student’s *t*-test. ^**^P < 0.01, ^***^P < 0.001, ^****^P < 0.0001.

We next evaluated the effects of the two G4-specific ligands on the replication of live virus. We first measured the effects of PDP, TMPyP4, TMPyP2 and remdesivir (as a positive control) on the cytotoxicity, viral RNA copies and viral titers in monkey kidney Vero E6 cells. Except for TMPyP2, addition of the other three compounds significantly reduced viral RNA copies and viral titers in Vero E6 cells in a dose-dependent manner (Fig. 4a–c; Supplementary Fig. S12a–f). More importantly, the antiviral activity of TMPyP4 is more potent than remdesivir, as evidenced by the maximal ~6-log_10_ vs 3-log_10_ viral RNA copies reduction in the Vero E6 cell lysate (Fig. 4a), ~7-log_10_ vs ~4-log_10_ viral RNA copies reduction in the Vero E6 cell culture supernatant (Fig. 4b), and ~6-log_10_ vs ~3-log_10_ viral titers reduction in the culture supernatant of Vero E6 cell (Fig. 4c). In addition, 10% cytotoxic concentration (CC_10_), half-cytotoxic concentration (CC_50_), half-maximal effective doses (EC_50_), 90% maximal effective doses (EC_90_) and selectivity index (SI) of the three compounds were determined. Compared with PDP (EC_50_ = 0.63 μM; EC_90_ > 2.00 μM; CC_10_ = 2.84 μM; CC_50_ = 4.90 μM; SI = 7.78) and remdesivir (EC_50_ = 1.33 μM; EC_90_ = 2.42 μM; CC_10_ = 1.04 μM; CC_50_ > 100 μM; SI > 75.19), TMPyP4 (EC_50_ = 8.87 μM; EC_90_ = 13.21 μM; CC_10_ = 727.7 μM; CC_50_ > 1000 μM; SI > 112.74) exhibited lower cytotoxicity and a higher SI (Fig. 4d). TMPyP4 also inhibited virus infection efficiently in human lung epithelial Calu-3 cells (Supplementary Fig. S13a–d). Consistently, TMPyP4 but not TMPyP2 could significantly suppress the protein levels of nucleoprotein in both Vero E6 and Calu-3 cells (Supplementary Fig. S13e). More importantly, TMPyP4 treatment could decrease the mRNA expression of pro-inflammatory cytokines and chemokines in Calu-3 cells (Supplementary Fig. S13f), suggesting that TMPyP4 may relieve SARS-CoV-2-induced inflammation. We further investigated whether TMPyP4 and remdesivir worked against SARS-CoV-2 in an additive or synergistic fashion, and found that TMPyP4 did not exhibit antiviral synergy with remdesivir (Supplementary Fig. S14).

**Fig. 5:**
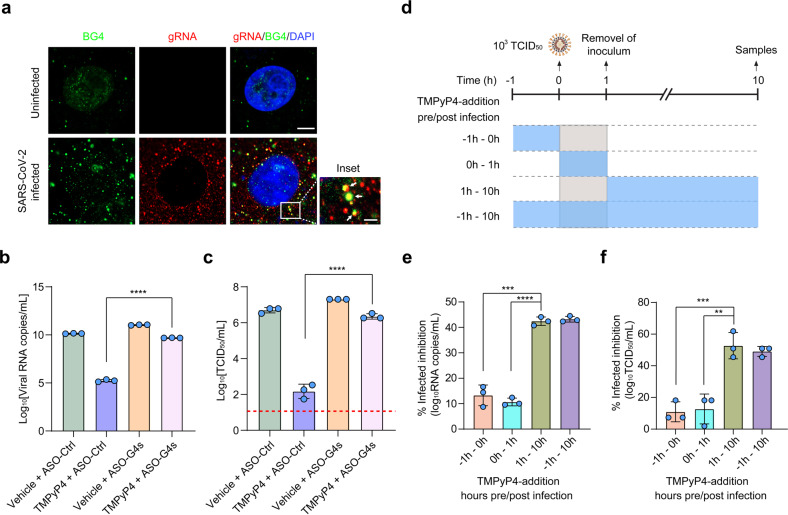
TMPyP4 inhibits viral infection by binding SARS-CoV-2 genomic G4s. **a** Immunofluorescence of BG4 and fluorescence in situ hybridization (FISH) for viral genomic RNA (gRNA) in Vero E6 cells with or without ARS-CoV-2 infection. Scale bars, 10 μm. In the inset of selected regions, the colocalized foci of gRNA (red) with BG4 (green) are indicated by white arrows. The scale bar of inset, 2 μm. **b**, **c** The Vero E6 cells treated with 100 µM TMPyP4 were pre-transfected with the ASOs targeting viral G4s, and then the cells were infected with SARS-CoV-2 (10^3^ TCID50 virus/mL). At 72 h post infection, viral RNA copies in cell culture supernatants (**b**) were detected by qRT-PCR. Viral titers (log_10_TCID_50_/mL) (**c**) were quantified by TCID_50_. Lower limit of detection for viral titers is indicated with a red dotted line. **d**–**f** Time-of-addition experiment of TMPyP4. The scheme shows the experimental design and the period of cell-drug incubation (**d**). Vero E6 cells were incubated with 100 μM TMPyP4 at the time points indicated. The cells were infected with SARS-CoV-2 (10^3^ TCID_50_ virus/mL), and the cell culture supernatants viral RNA copies (**e**) and viral titers (**f**) were quantified by qRT-PCR and TCID_50_. Data are shown as means ± SEM of three independent experiments, two-tailed Student’s *t*-test. ^**^P < 0.01, ^***^P < 0.001, ^****^P < 0.0001.

We next investigated whether TMPyP4 inhibited SARS-CoV-2 infection by binding the SARS-CoV-2 genomic RNA G4s. First, we stained the viral RNA G4s by BG4 antibody in SARS-CoV-2-infected Vero E6 cells and observed co-localization between BG4 fluorescence and viral genomic RNA (gRNA) (Fig. 5a), suggesting the formation of viral G4 structures during SARS-CoV-2 replication. Antisense oligonucleotides (ASOs) are used to ‘open’ G4 structures^26,27^, thus we designed a series of ASO complementary to viral G4 sequences to unfold viral G4 structures (Supplementary Fig. S15a). We observed that the antiviral activity of TMPyP4 was significantly inhibited by ASO targeting viral G4s (Fig. 5b, c; Supplementary Fig. S15b), indicating that TMPyP4 inhibits virus infection by targeting viral G4s. Furthermore, time-of-addition assays suggested that TMPyP4 functioned at a stage post SARS-CoV-2 entry (Fig. 5d–f), consistent with its putative antiviral mechanism by targeting viral G4s. Worth to note, TMPyP4 treatment significantly decreased the viral loads in Vero E6 cells pre-infected with SARS-CoV-2 for 12 h or 24 h (Supplementary Fig. S16a, b), suggesting the potential therapeutic effect of TMPyP4 for COVID-19.

### TMPyP4 suppresses viral infection and lung inflammation in animal models of SARS-CoV-2 infection

**Fig. 6:**
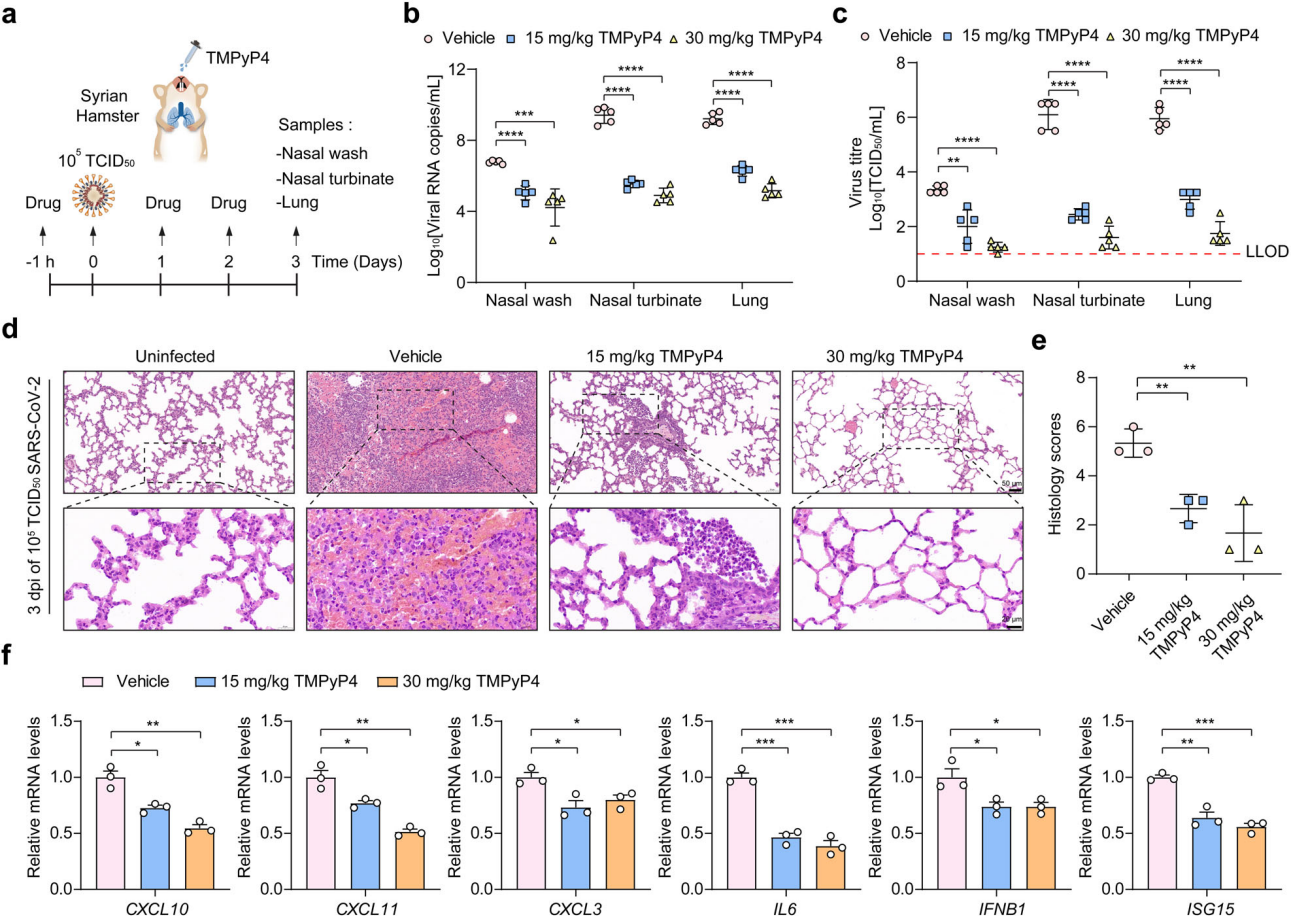
Antiviral activity of TMPyP4 in SARS-CoV-2-infected hamster. **a** Schematic of SARS-CoV-2 infected hamster model. Hamsters infected with SARS-CoV-2 (10^5^ TCID_50_ virus/hamster) were treated with vehicle (*n* = 5), 15 mg/kg (*n* = 5) or 30 mg/kg (*n* = 5) TMPyP4 for consecutive 3 days, with the first dose given at 1 h before infection with SARS-CoV-2. **b**, **c** Viral RNA copies (**b**) and viral titers (**c**) in the hamster nasal wash, nasal turbinate and lung tissues of the TMPyP4-treated groups relative to vehicle controls, determined by qRT-PCR and TCID_50_ at day 3 after infection. LLOD for viral titers is indicated with a red dotted line. **d** The representative images of hamster lung histopathological changes at day 3 after infection. The corresponding higher-magnification images were shown. See Supplementary Fig. S20 for whole-lung tissue scan images of all hamsters. **e** Pathological severity scores in hamsters with SARS-CoV-2 infection. **f** Representative chemokine and cytokine assessment of the lung tissues (*n* = 3) of the indicated groups, as detected in lung tissue homogenate at day 3 after infection. Data are shown as means ± SEM, two-tailed Student’s *t*-test. ^*^P < 0.05, ^**^P < 0.01, ^***^P < 0.001, ^****^P < 0.0001.

**Fig. 7:**
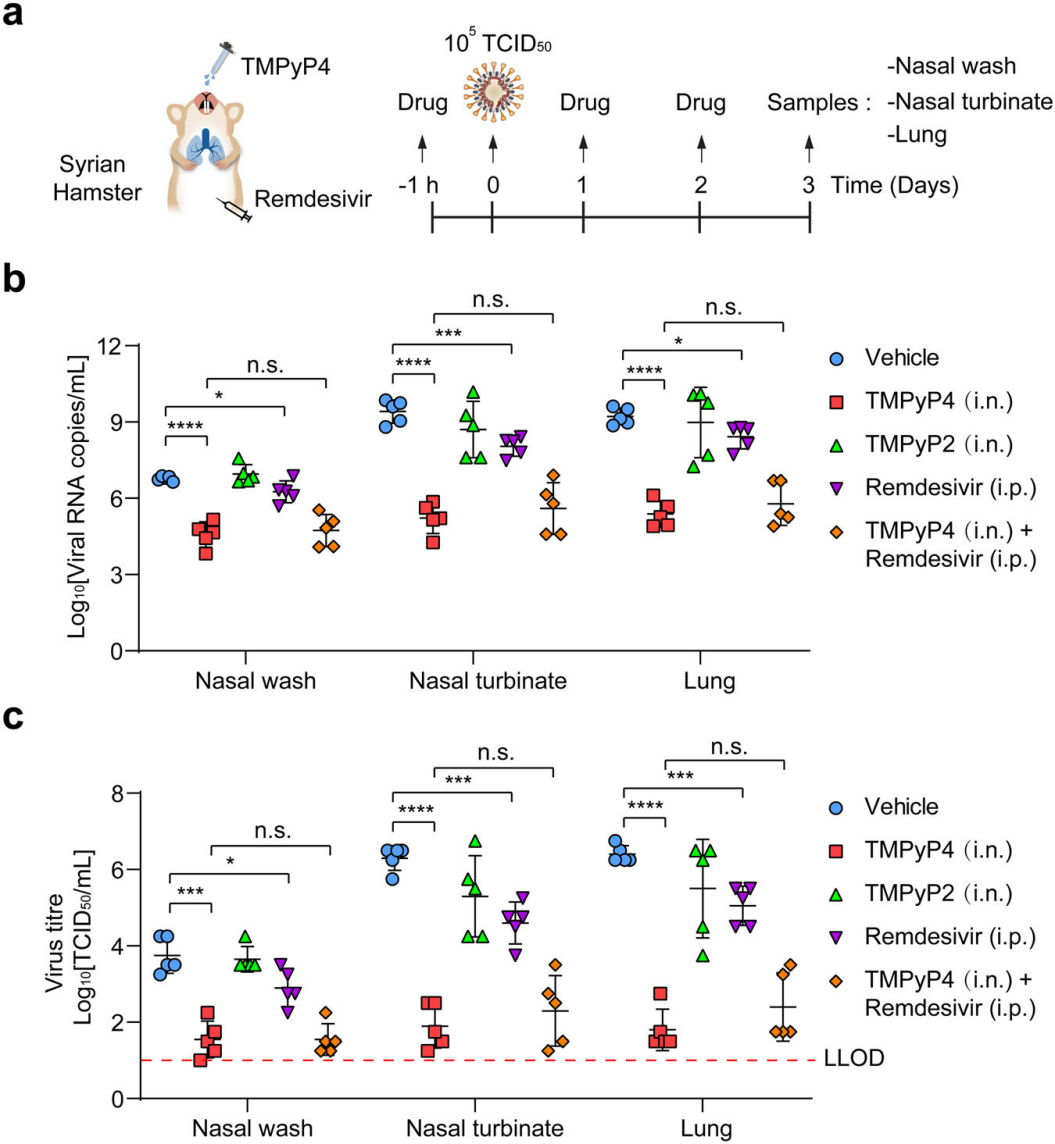
The antiviral activity of TMPyP4 is more potent than remdesivir in SARS-CoV-2-infected hamster. **a** Schematic of SARS-CoV-2-infected hamster model. Hamsters infected with SARS-CoV-2 (10^5^ TCID_50_ virus/hamster) were treated with vehicle (*n* = 5), 15 mg/kg TMPyP4 (*n* = 5), 15 mg/kg TMPyP2 (*n* = 5), 15 mg/kg remdesivir (*n* = 5) and 15 mg/kg remdesivir combined with 15 mg/kg TMPyP4 (*n* = 5) for consecutive 3 days, with the first dose given at 1 h before infection with SARS-CoV-2. **b**, **c** Viral RNA copies (**b**) and viral titers (**c**) in the hamster nasal wash, nasal turbinate and lung tissues of the indicated groups, determined by qRT-PCR and TCID_50_ at day 3 after infection. Data are shown as means ± SEM, two-tailed Student’s *t*-test. n.s. not significant. ^*^P < 0.05, ^***^P < 0.001, ^****^P < 0.0001.

To identify whether TMPyP4 is suitable for in vivo antiviral studies, we conducted pharmacokinetic and toxicity experiments in golden Syrian hamster model. A prior study has shown that TMPyP4 shows no noticeable toxicity in the animals for short-term treatment^28^, while the long-term side effects are undetermined. As a novel drug delivery strategy for COVID‐19 therapeutics and prevention, intranasal (i.n.) drug administration is safe, easy to apply, and could provide rapid onset of action, offering multiple advantages in comparison to traditional routes^29–31^. Thus, we adopted the i.n. administration route of TMPyP4 to investigate the pharmacokinetics and toxicity of TMPyP4 in vivo. When i.n. administered with TMPyP4 (30 mg/kg), C_max_ (17.88 μg/mL) was observed at 1 h post-dosing, followed by a gradual decrease as observed from the plasma concentration-time profile (Supplementary Fig. S17a, b). TMPyP4 displayed a half-life (T_1/2_) of 6.36 h and area under the curve (AUC) values of 167.32 h⋅μg/mL (Supplementary Fig. S17a). On the basis of the EC_50_/EC_90_ values from Vero E6 cells, a single i.n. dose of 30 mg/kg/day TMPyP4 maintained the plasma levels at EC_50_ and EC_90_ for ~10 and 6 h (Supplementary Fig. S17b), indicating that TMPyP4 is suitable for COVID-19 treatment in vivo. And we found that i.n. administration of TMPyP4 (30 mg/kg) once daily for 7 consecutive days lead to no noticeable toxicity in the hamsters (Supplementary Figs. S18 and S19). For in vivo antiviral assays, the hamsters were i.n. inoculated with SARS-CoV-2 (10^5^ TCID_50_ virus/hamster), and treated with vehicle (control) and TMPyP4 (15 mg/kg or 30 mg/kg i.n. starting at 1 h prior to virus inoculation and continuing until 3 days post infection) (Fig. 6a). We found that TMPyP4 treatment significantly decreased the mean viral loads (log_10_viral RNA copies/mL and log_10_TCID_50_/mL) in the nasal wash, nasal turbinate and lung tissues by 2–4 log_10_ (Fig. 6b, c). On day 3 post infection, the vehicle-treated hamsters showed moderate inflammatory cell infiltration and alveolar septal thickening, while TMPyP4 treatment could significantly ameliorate lung damage (Fig. 6d, e; Supplementary Fig. S20). Consistently, we found that TMPyP4 reduced mRNA expression of inflammatory-associated genes in the lungs (Fig. 6f), suggesting that TMPyP4 could ameliorate lung damage by affecting SARS-CoV-2-induced host immune response. We also compared the in vivo antiviral effects of remdesivir (15 mg/kg, intraperitoneal, i.p.), TMPyP4 (15 mg/kg, i.n.), TMPyP2 (15 mg/kg, i.n.) and combination of TMPyP4 (15 mg/kg, i.n.) and remdesivir (15 mg/kg, i.p.) (Fig. 7a). TMPyP4 exhibited more potent in vivo antiviral activity than remdesivir, as evidenced by the maximal ~3-log_10_ vs 1-log_10_ viral RNA copies reduction in the nasal wash, nasal turbinate and lung tissues (Fig. 7b) and ~3.5-log_10_ vs ~1.5-log_10_ viral titers reduction in the nasal wash, nasal turbinate and lung tissues (Fig. 7c). Consistent with in vitro antiviral assays, TMPyP4 did not exhibit antiviral synergy with remdesivir in hamsters (Fig. 7b, c). TMPyP4 treatment significantly decreased the viral loads in the nasal wash, nasal turbinate and lung tissues from the hamsters pre-infected with SARS-CoV-2 for 24 h (Supplementary Fig. S21a–c), and could ameliorate lung damage (Supplementary Fig. S21d). In hACE2-KI/IOZ C57BL/6 mouse model, we also found that TMPyP4 treatment significantly decreased the viral loads in the nasal turbinate and lung tissues of SARS-CoV-2-infected mouse (Supplementary Fig. S22a–c), consistent with the results in hamster. Collectively, these results indicate that i.n. administration of TMPyP4 inhibits SARS-CoV-2 replication, resulting in reduced viral loads and lung lesions.

## Discussion

In spite of great efforts around the globe, treatment options for COVID-19 are still limited. At present, different strategies have been employed against SARS-CoV-2 infection, including direct inhibition of virus replication by targeting viral protease, RdRP or blocking the interactions between viral spike protein with the host protein ACE2 or TMPRSS2^22^, while we provided an alternative therapeutic strategy focusing on targeting functional secondary structures within SARS-CoV-2 genome. Here, we have defined the mechanism by which RNA G4s, identified within SARS-CoV-2 genome, suppress the translation of viral proteins and viral RNA replication, thereby regulating the life cycle of SARS-CoV-2 (Supplementary Fig. S23). In addition, G4 ligands targeting the SARS-CoV-2 G4s show powerful antiviral activity and are promising therapeutic candidates for COVID-19.

In recent years, G4s have been discovered in almost all organisms including many viruses and are involved in regulating the viral life cycle. The formation of G4s in HIV genome can regulate viral transcription^32^. Zaire ebolavirus (EBOV) genomic G4s have shown to suppress the expression of L gene, resulting in the prevention of viral replication^33^. Thus, G4s have been considered as potential targets for antiviral strategies. Our prior study has reported an RNA G4 in SARS-CoV-2 genome, whose formation can inhibit the expression of nucleocapsid phosphoprotein^18^. However, there are still many other undefined G4s in SARS-CoV-2 genome, and whether G4s can act as anti-SARS-CoV-2 therapeutic targets are still confused. Here, we confirmed the existence of six additional G4s in positive- or negative-sense strands of SARS-CoV-2. Furthermore, we found that G4s formation can inhibit both the viral replication and translation processes. These findings highlighted that G4s in SARS-CoV-2 genome have dual-function of inhibiting the viral RNA synthesis as well as viral protein expression, suggesting the great potential of G4s as anti-SARS-CoV-2 therapeutic targets. More importantly, due to the extensive existence and regulatory roles of G4s in the coronavirus genome, the development of drugs targeting G4s may be of paramount importance for the treatment and/or prophylaxis of the current pandemic but potentially also for future and unavoidable outbreaks of human pathogenic coronaviruses.

TMPyP4 was used as G4-specific stabilizer because of its suitable physical properties for stacking with the G tetrads^34^. Due to the photodynamic properties and inhibition of telomerase at low micromolar concentrations, TMPyP4 has been identified as an attractive candidate for anticancer drug development^35,36^. Recently, several studies have shown the antiviral activity of TMPyP4 against multiple viruses by targeting G4s in cell-based assays^37,38^. However, whether TMPyP4 can effectively suppress the viral infection in vivo has not been investigated. In this study, we demonstrate that TMPyP4 treatment significantly inhibits SARS-CoV-2 infection in cell-based assays. In addition, intranasal administration of TMPyP4 significantly inhibits SARS-CoV-2 replication and relieves lung lesions with no observable toxicity in two animal models. More importantly, the anti-SARS-CoV-2 activity of TMPyP4 is more potent than remdesivir in vitro and in vivo, as evidenced by the lower viral loads of SARS-CoV-2 after TMPyP4 treatment compared with remdesivir. To our knowledge, it is the first proof for the antiviral effects of TMPyP4 in vivo, and these findings support the clinical application potential of TMPyP4 as a broad-spectrum antiviral agent against multiple viruses, especially SARS-CoV-2.

There are still limitations in this study. First, cellular G4s broadly influence many biological processes of transcription, translation and genome stability etc.^12^, thus the off-target of TMPyP4 may cause side effects. Recently, a growing number of studies proposed that during infection the virus extensively replicates its genome to create a large number of viral G4s considerably exceeding that of cellular G4s, which may compensate for a limited intra-G4 specificity of a ligand^15,39,40^, e.g., there is a sharp increase in the number of viral G4s during herpes simplex virus-1(HSV-1) replication^41^. Consistently, we found that SARS-CoV-2 infection in Vero E6 cells may create hundreds copies of viral RNA G4 targets (as shown by immunostaining by a G4-specific antibody BG4 in Fig. 5a). Therefore, because the amount of viral G4s may outstand that of cellular G4s by several logs per cell during viral infection and the antiviral effects of TMPyP4 can be achieved at low micromolar concentrations, the off-target effects might be reduced in a large part. It is notable that unfolding the viral G4s by ASOs significantly suppressed the antiviral effects of TMPyP4, suggesting that inhibition of SARS-CoV-2 infection following TMPyP4 administration is mainly due to the binding of TMPyP4 to viral G4s, but not the host G4s. Besides, TMPyP4 exhibited no observable toxicity at an effective antiviral concentration both in vitro and in vivo. Despite the long-term side effects are undetermined, we have reasons to believe that TMPyP4 has the potential to be a short-term treatment of COVID-19 for 1–2 weeks, as COVID-19 is an acute disease. And the compounds specifically targeting viral G4s, but not host G4s, deserve to be further investigated. Second, although we have demonstrated the regulatory effects of G4s on the replication and translation processes of SARS-CoV-2, the roles of G4s in SARS-CoV-2 life cycle are not fully elucidated. The transmission of asymptomatic carriers of SARS-CoV-2 infection is still unclear, making the mechanism underlying SARS-CoV-2 latency to be illustrated. Several recent studies have shown that G4s can regulate the viral incubation period and immune evasion^26,42^. Whether SASR-CoV-2 G4s are involved in regulating viral incubation period deserves further investigation.

In summary, we demonstrate the important roles of G4s in the viral life cycle of SARS-CoV-2, which might be a novel target for the design of more potent drugs against COVID-19. Moreover, our in vitro and in vivo studies have identified that TMPyP4 can bind to SARS-CoV-2 G4s and has even better antiviral effects than remdesivir. TMPyP4 can be a short-term anti-SARS-CoV-2 agent that should be accelerated into clinical trials for COVID-19. By providing an alternative tactic for COVID-19 therapy by targeting SARS-CoV-2 genomic secondary structures, this work would facilitate the rational design and synthesis of drug candidates with high selectivity toward the new targets.

## Materials and methods

### Oligonucleotides and reagents

All RNA oligonucleotides were synthesized by Shanghai Sangon Biological Engineering Technology & Services (Shanghai, China). The oligonucleotide sequences were described in Supplementary Table S3. Concentrations of the oligomers were determined by measuring the absorbance at 260 nm after melting. All DNA or RNA samples were heated at 95 °C for 5 min, and then slowly cooled to room temperature unless otherwise indicated. TMPyP4 was purchased from Tokyo Chemical Industry (TCI). PDP was a generous gift from Prof. S. Wang (Wuhan University, Wuhan, China). NMM was purchased from Sigma-Aldrich. All chemicals were used without further purification.

### Fluorescence assays

For NMM fluorescence assays, the DNA or RNA oligonucleotide samples pre-heated at 95 °C for 10 min were slowly cooled to room temperature before co-incubated with NMM. Then, 0.6 μM NMM and 0.3 μM samples were co-incubated 4 h. The NMM fluorescence assays were performed by using JASCO FP-6500 spectrofluorometer at room temperature.

### Circular dichroism (CD) measurements

For CD spectra and CD melting experiments, the DNA or RNA oligonucleotide samples pre-heated at 95 °C for 10 min were slowly cooled to room temperature, and then were incubated at 4 °C for 24 h. The samples were detected by JASCO J-810 spectropolarimeter equipped with a temperature-controlled water bath. In CD melting experiments, versus temperatures were collected at a heating rate of 1 °C/min.

### Nondenaturing polyacrylamide gel electrophoresis experiments

Native gel electrophoresis was performed by acrylamide gel (15%) and run at room temperature, 1× TB buffer containing 10 mM KCl and was silver stained. About 2 μM RNAs were loaded on the gel.

### NMR spectroscopy

1H NMR spectra were carried out on a Bruker Avance 600 MHz NMR Spectrometer. The RNA samples were dissolved in 10 mM phosphate-buffered saline (PBS) (pH 7.0) containing 100 mM KCl and 10% D_2_O at a final concentration of 0.3 mM in strand.

### Viruses and cells

The SARS-CoV-2 (BetaCoV/Beijing/IME-BJ05-2020) was propagated and titrated on African green monkey kidney epithelial cells (Vero E6) (CRL1586, ATCC, USA) and human lung epithelial Calu-3 cells (HTB-55, ATCC, USA). The human cervical carcinoma cell Hela and the human embryonic kidney cell line HEK293T were a generous gift from Dr. Qian Jin (Beijing Institute of Lifeomics, Beijing, China). All these cell lines were maintained in high-glucose Dulbecco’s modified Eagle’s medium (DMEM; HyClone, USA) supplemented with 10% fetal bovine serum (FBS; HyClone, USA), 100 U/mL penicillin, and 100 μg/mL streptomycin. The cells were incubated at 37 °C in a humidified incubator containing 5% CO_2_.

### In vitro translation (IVT) assays

To generate the SARS-CoV-2 transcripts with P4 G4 site for the IVT assays, pGEM-T esay vector constructs encoding the SARS-CoV-2 genome sequences (nucleotides 3344–3643) with P4 G4 site or mutant G4 site fused to a Flag tag at the C-terminus were linearized with SpeI. For IVT assays, pGEM-T esay vector was transcribed and translated using TNT^®^ Quick Coupled Transcription/Translation System (Promega). Lysates were subjected to SDS-PAGE and autoradiography. IVT assays were also performed in the presence or absence of PDP or TMPyP4.

### Immunofluorescence assays

Cells were washed twice in 1× PBS, fixed with 4% paraformaldehyde for 10 min, permeabilized with 0.5% Triton X-100-PBS for 15 min, and then incubated after with 100 mg/mL RNase A for 1 h at 37 °C, and then blocked for 30 min with 3% BSA-0.2% Triton X-100-PBS. Furthermore, cells were incubated with primary antibody for 16 h at 4 °C. BG4 specific antibody (anti-DNA/RNA G-quadruplex (and related small molecules) (BG4) Standard Size Ab00174-1.6) was purchased from absolute antibody (Wilton, UK). Fluorescent secondary antibodies were used in a 1:1000 dilution for 45 min at 37 °C. DAPI was incubated for 2 min in the dark before taken images with confocal laser-scanning microscope.

### RNA replication assays

Cell-based RNA replication assay system was applied^43–45^. The plasmids nsp12, nsp7 and nsp8 were used to express codon-optimized Flag-nsp12, Flag-nsp7 and Flag-nsp8, respectively, all of which contain a Flag tag at the C-terminus. To generate the SARS-CoV-2 transcripts with P4 G4 site for RNA replication assays, 5’ untranslated region (UTR)-P4 G4 -3’ UTR was first synthesized, then inserted into the pGEM-T esay vector. And the SARS-CoV-2 RNA with P4 G4 site was produced using T7 RNA polymerases from a standard in vitro transcription kit.

HEK293T cells were transfected with the RNA with P4 G4 site, nsp12, nsp7, and nsp8 plasmids at the ratio of 1:10:30:30. At 12 h post transfection, cells were re-seeded in 24-well plates (4 × 10^4^/well) and treated with PDP or TMPyP4. After 48 h incubation, cells were collected and measured for RNA levels by qRT-PCR assays.

### Antisense oligonucleotides assays

All the locked nucleic acid (LNA) antisense oligonucleotides (ASOs) targeting the SARS-CoV-2 G4s were synthesized by Shanghai Sangon Biological Engineering Technology & Services (Shanghai, China). The sequences were described in Supplementary Table S3. For ASOs treatment, Vero E6 cells were pre-transfected with a mixture of 17-mer ASOs targeting P2, P3, P4, P6, P10, P13 and P14 G4s or a random 17-mer ASO for 6 h prior to virus infection and TMPyP4 treatment. At 72 h post infection, cell supernatant was collected for qRT-PCR and viral titer assays.

### Time-of-addition experiment of TMPyP4

A time-of-drug-addition assays were performed to investigate the stage of SARS-CoV-2 life cycle that TMPyP4 (100 μM) interferes with. Vero E6 cells (4 × 10^5^ cells/well) infected with 103 TCID_50_ of SARS-CoV-2 were treated with TMPyP4 or vehicle at time points indicated, followed by incubation at 37 °C in 5% CO_2_. At 10 h post infection, cell supernatant was collected for qRT-PCR and viral titer assays.

### Statistical analyses

All statistical analyses were performed by SPSS 13.0 software (Chicago, IL, USA) and GraphPad Prism software (La Jolla, CA, USA). Student’s *t*-test was used to compare the quantitative data between two groups. Data are presented as means ± SEM, and all experiments were performed in at least 3 replicates. Statistical differences are indicated as follows: ^*^P < 0.05, ^**^P < 0.01, ^***^P < 0.001, ^****^P < 0.001. n.s., not significant.

## Supplementary information

Supplementary Information
