# Supplementary material for: RNA G-quadruplex formed in SARS-CoV-2 used for COVID-19 treatment in animal models
Source: Cell Discov. 2022 Sep 6;8:86. doi: 10.1038/s41421-022-00450-x (PMC9447362; doi:10.1038/s41421-022-00450-x)
Supplement: Supplementary file 1 — Supplementary Information [file 41421_2022_450_MOESM1_ESM.pdf]

Supplementary Materials for

RNA G-quadruplex formed in SARS-CoV-2 used for COVID-19 treatment in animal models

Authors

Geng Qin,[1,2]† Chuanqi Zhao,[1,2]† Yan Liu,[3]† Cheng Zhang,[3,4]† Guang Yang,[3] Jie Yang,[1,2] Zhao Wang,[1,2] Chunyu Wang,[5] Changchun Tu,[3] Zhendong Guo,[3] Jinsong Ren,[1,2] and Xiaogang Qu*[,1,2]

Affiliations

[1]Laboratory of Chemical Biology and State Key Laboratory of Rare Earth Resource Utilization, Changchun Institute of Applied Chemistry, Chinese Academy of Sciences, Changchun, Jilin, China
[2]University of Science and Technology of China, Hefei, Anhui, China
[3]Changchun Veterinary Research Institute, Chinese Academy of Agricultural Sciences, Changchun, Jilin, China
[4]Hebei Agricultural University, College of Veterinary Medicine, 2596 Lucky South Street, Baoding, Hebei, China
[5]State Key Laboratory of Supramolecular Structure and Materials, Jilin University, Changchun, Jilin, China
†These authors contributed equally to this work

*Corresponding author: Xiaogang Qu.
Email: xqu@ciac.ac.cn

This PDF file includes:

Supplementary information text
Supplementary materials and methods
Supplementary Tables S1 to S3
Supplementary Figs. S1 to S23

Supporting information text

Table S1. Characteristics of the 18 putative G-quadruplexes.

Table S2. The stability and thermodynamic parameters of 14 putative G-quadruplexes.

Table S3. Oligonucleotides and primers in this study.

Fig. S1. Identification and selection of G-quadruplexes (G4s) in SARS-CoV-2 genome.

Fig. S2. Screening the PQSs in SARS-CoV-2 genome by fluorescence turn-on assays.

Fig. S3. The structure and stability of PQS candidates.

Fig. S4. The analysis of the conservation of PQS candidates.

Fig. S5. Visualization of the PQSs in six human coronaviruses.

Fig. S6. The number of PQSs in seven human coronaviruses.

Fig. S7. The formation of P4 G4 in vitro.

Fig. S8. Chemical structures of TMPyP2.

Fig. S9. The formation of P4 G4 inhibits the protein expression.

Fig. S10. TMPyP2 treatment does not inhibit the RNA translation and replication.

Fig. S11. TMPyP4 treatment suppresses the RNA translation and replication by targeting the other G4s in SARS-CoV-2.

Fig. S12. The cytotoxicity and antiviral activity of TMPyP4 and TMPyP2 treatment in Vero E6 cells.

Fig. S13. The cytotoxicity and antiviral activity of TMPyP4 in Calu-3 cells.

Fig. S14. TMPyP4 did not exhibit antiviral synergy with remdesivir.

Fig. S15. ASO suppresses the effect of TMPyP4 on P4-EGFP SARS-CoV-2 lentivirus infection.

Fig. S16. Therapeutic treatment of TMPyP4 inhibits viral replication in Vero E6 cells.

Fig. S17. Primary pharmacokinetic (PK) evaluation of TMPyP4 in hamsters.

Fig. S18. Intranasal administration of TMPyP4 (30 mg/kg) once daily for 7 consecutive days show no noticeable toxicity in the lung tissues of hamsters.

Fig. S19. Intranasal administration of TMPyP4 (30 mg/kg) once daily for 7 consecutive days show no noticeable toxicity in the liver and kidney tissues of hamsters.

Fig. S20. Scanning images of whole lung tissue sections.

Fig. S21. Antiviral activity of TMPyP4 in the hamster pre-infected with SARS-CoV-2.

Fig. S22. Antiviral activity of TMPyP4 in hACE2 transgenic mouse model of SARS-CoV-2 infection.

Fig. S23. The regulatory mechanism of G4s in SARS-CoV-2 life cycle.

Supplementary materials and methods

Bioinformatics analysis

The complete genome sequences of 7 known human coronavirus (HCoV-229E、HCoV-OC43、HCoV-NL63、HCoV-HKU1、SARS-CoV-1, MERS-CoV and SARS-CoV-2) were retrieved from the NCBI Genome database (https://www.ncbi.nlm.nih.gov/genomes). The FASTA sequences of the SARS-CoV-2 genomes were then used for the prediction of the putative G4-forming sequences (PQSs) with ImGQfinder[1], Pqsfinder[2], QGRS-mapper[3] and G4Catchall[4]. The search was based on the following algorithm:

$G_{\geq 2}N_{1\text{-}15}G_{\geq 2}N_{1\text{-}15}G_{\geq 2}N_{1\text{-}15}G_{\geq 2}$

where G corresponds to guanine and N corresponds to any base including guanine. Though the SARS-CoV-2 is a positive-sense RNA virus, we also sought to find the PQSs in the negative-sense strand of the viral genome. The ImGQfinder (http://imgqfinder.niifhm.ru/), Pqsfinder (https://pqsfinder.fi.muni.cz/), QGRS-mapper (https://bioinformatics.ramapo.edu/QGRS/analyze.php) and G4CatchALL (http://homes.ieu.edu.tr/odoluca/G4Catchall/) were run using their online tools.

For evaluating the G4 folding capabilities by the consecutive G over consecutive C ratio (cGcC), G4Hunter (G4H) and G4 neural network (G4NN) scores, the G4RNA screener (http://scottgroup.med.usherbrooke.ca/G4RNA_screener/) were used[5].

cGcC was implemented to address the issue of competition in between G4 and Watson-Crick based structures. The presence of cytosine runs in the vicinity of a potential G4 was demonstrated to be an important feature to consider in the identification of potential G4 since the base pairing of those C runs with G runs involved in the potential G4 can hinder its formation. This score varies on a logarithmic scale by its ratio nature[6]. In this study, PQSs with a ratio value superior to the arbitrary threshold of 4.5 were considered as being accessible under conditions favouring G4 folding.

G4H was designed in a similar way to the cGcC score but was built to analyze DNA sequences. We demonstrated its relevance in RNA as well. It attributes an

increasing positive score to each contiguous G and a negative counterpart for contiguous C. The sequence is then scored by the average of the values[7]. In this study, PQSs with a ratio value superior to the arbitrary threshold of 0.9 were considered as being accessible under conditions favouring G4 folding.

Sequences of the G4RNA database were converted into vectors of their trinucleotide content to train an artificial neural network. This artificial neural network, G4NN, evaluates the similarity of a given sequence to known G-quadruplexes and reports it as a score between 0 and 1[8]. In this study, PQSs with a ratio value superior to the arbitrary threshold of 0.5 were considered as being accessible under conditions favouring G4 folding.

For the analysis of the conservation of the 7 PQS sites across strains, 3969 complete genomic sequences of SARS-CoV-2 genome were retrieved from the University of California Santa Cruz (UCSC) genome browser, and the graphical representation of the aligned sequences was generated using the WebLogo 3 software program (http://weblogo.threeplusone.com/)[9].

For the analysis of the conservation of the 7 PQS sites throughout the human coronavirus family, 7 known human coronavirus genomic sequences were retrieved from the University of California Santa Cruz (UCSC) genome browser, and the graphical representation of the aligned sequences was generated using the WebLogo 3 software program.

Thermodynamic parameters obtained by melting analysis

In order to calculate thermodynamic parameters, including the enthalpy change (ΔH^o), the entropy change (ΔS^o), and the free energy change (ΔG^o) for intramolecular G4 formation, the melting curves were fit to the theoretical equation. ΔH^o was the slope of the ln Ka versus 1/T plot according to the equation ln Ka = $-(\Delta H^o)$, /RT) +ΔS^o /R, where ΔS^o was the entropy change that was calculated according to the y axis intercept. The free energychange ($\Delta G^o{}_{25}$) was calculated from the standard Gibbs's equation, $\Delta G^o{}_{25} = \Delta H^o - T\Delta S^o$.

Stopped-flow experiments

Fluorescence stopped-flow experiments were carried out by using an SX20

Stopped-Flow Spectrometer. In these assays, NMM treated samples (NMM/F-P4-WT or NMM/F-P4-Mut in 10 mM Tris-HCl buffer, 0 mM KCl, pH = 7.2) were mixed with K^+-containing buffer (10 mM Tris-HCl buffer, 200 mM KCl, pH = 7.2). Fluorescence changes were monitored at a wavelength of 661 nm with an excitation wavelength of 395 nm.

Simultaneous fluorescence in situ hybridization (FISH) and immunofluorescence.

One set of Stellaris FISH probes, targeting SARS-CoV-2 RNA were obtained from Shanghai Sangon Biological Engineering Technology & Services (Shanghai, China). Cells grown on chambered dishes were fixed with 4% paraformaldehyde overnight in the ABSL3 laboratory. Cells were then permeabilized with 70% ethanol for at least 2 h and subjected to FISH and immunofluorescence. Briefly, cells were incubated with BG4 specific antibody (1:400) and viral RNA FISH probes (1:1000) in Hybridization Buffer consisting of 20 μg/mL fish sperm DNA (fsDNA), 50 μg/mL heparin, 50% Deionized formamide, 0.1% SDS, 5 × Denhardt's and 5 × SSC (PH = 7.0) for at least 4 h at 37 ℃. Slides were then washed three times and then incubated with secondary antibodies for 1 h at 37 ℃. Finally, slides were washed three times and incubated with DAPI for 2 min to stain nuclear DNA in the dark before taken images with confocal laser-scanning microscope. The probes were described in **Table S3**.

Vectors construction and transfection

A reporter vector with the pLV-EGFP-N backbone (Inovogen Tech. Co., Beijing, China) was generated to encode the SARS-CoV-2 genome sequences with P1, P2, P3 and P6 G4 sites. The SARS-CoV-2 genome sequences (nucleotides 3,344-3,643) with P4 G4 site were PCR-amplified and subcloned into pCAG-Flag vector and pLV-EGFP-N backbone (Inovogen Tech. Co., Beijing, China). And the pCAG-Flag is a generous gift from Prof. Peihui Wang (Cheeloo College of Medicine, Shandong University, Jinan). By using the Stratagene QuikChange Site-Directed Mutagenesis kit, we point mutated the P4 G4 site.

For viral packaging and infection were performed as previously described[10]. Briefly, the pLV-EGFP-N along with viral packaging plasmids (PMD and SPA) were

transfected into human embryonic kidney 293T cells using Lipofectamine 2000 (Invitrogen, USA), following the manufacturer's instructions. Virus supernatant was harvested after 48 hr, filtered through a 0.45 μM filter, and incubated on target cells for 6 hr at a 1:10 dilution with 8 μg/mL polybrene. For transfection, the cells were transfected with the indicated vectors using Lipofectamine 2000 (Invitrogen, CA, USA) following the manufacturer's protocol. The transfected cells were harvested after 48 to 72 h.

Western blotting assays

For protein analysis of whole-cell lysates, cells were lysed in RIPA (CWBIO, Beijing, China) buffer. Total proteins (10 - 20 μg) were re-suspended in Laemmli buffer (63 mM Tris-HCl, 10% glycerol, 2% SDS, 0.0025% bromophenolblue, pH 6.8) and electrophoresed on SDS-polyacrylamide gels. Then, proteins were transferred to polyvinylidene difluoride membrane. After an incubation with antibodies specific for nucleocapsid phosphoprotein (40143-R001; Sinobiology, China), GFP (AF1483; Beyotime, Shanghai, China), Flag (bs-0965R; Bioss, Beijing, China) or GAPDH (#CW0098M; CWBIO, Beijing, China), the blots were incubated with anti-mouse (#CW0102M; CWBIO, Beijing, China) or anti-rabbit (#CW0103M; CWBIO, Beijing, China) secondary antibodies conjugated to horseradish peroxidase (HRP). The immunoreactive bands were detected using SuperSignal™ West Pico chemiluminescent substrate kit (Thermo Fisher Scientific, Waltham, MA, USA) and Western blotting detection system (BioRad, Hercules, CA, USA). The GAPDH was used as a loading control for western blotting assays.

Quantitative real-time polymerase chain reaction (qRT-PCR) assays

Total RNAs were extracted from cultured cells or tissues using RNApure tissue&cell kit (CWBIO, China) according to the manufacturer's protocol. The cDNAs were synthesized from 500 ng of total RNAs using PrimeScript RT reagent kit (Takara, Japan) according to the manufacturer's protocol. The qRT-PCR assays were performed using SYBR FAST qPCR kit (Kapa Biosystems, USA) at the BioRad IQ5 real-time PCR system (BioRad, Hercules, CA, USA). The relative expression levels of RNAs were calculated using the comparative C_t method. Melting curve analyses

were performed on all PCRs to rule out the non-specific amplification. The primers were described in **Table S3**.

Cell viability assays

Cell viability of Vero E6 cells with PDP or TMPyP4 treatment was assessed using SRB assay/sulforhodamine B assay kit (ab235935; Abcam, USA) according to the manufacturer's protocol, and the details were described in our previous study[11].

Evaluation of antiviral activities of TMPyP4 and PDP

The in vitro antiviral studies were carried out in an animal biosafety level 3 (ABSL3) in Changchun Veterinary Research Institute, Chinese Academy of Agricultural Sciences. To evaluate the antiviral efficacy of TMPyP4 and PDP, Vero E6 cells were cultured overnight in 6-well cell-culture petridish with a density of 10^6 cells/well. Cells were pre-treated with the different doses of the indicated antivirals for 1 h, and the virus (10^3 or 10^2 $TCID_{50}$) was subsequently added to allow infection for 1 h. Then, the virus-drug mixture was removed and cells were further cultured with fresh drug-containing medium. At 72 h post infection, the cell supernatant and lysates were collected for further quantification analysis. For investigating the antiviral effect of TMPyP4 therapeutic treatment, cells were pre-infected with the virus (10^3 $TCID_{50}$) for 12 or 24 h, and 100 μM TMPyP4 was subsequently added.

Viral infection and viral titer assays For viral infection, cells were incubated with virus for 1 h at 37 ℃ at the indicated $TCID_{50}$. Then, the excess virus inoculum was removed by washing three times with PBS. For viral titer assays, cells were seeded in 96-well plates at a density of 1 × 10^4 per well. On the next day, the cells were inoculated with serially diluted viruses (10^{-1}~10^{-12} fold) for 1 h at 37 ℃. The excess virus inoculum was removed by washing three times with PBS. Then, 200 μl of maintenance medium (DMEM/2% FBS) was added to each well, and the cells were further cultured for 3–5 days. The cells demonstrating the expected cytopathic effect were observed daily, and the $TCID_{50}$ value was calculated with the Reed-Muench method.

Viral RNA extraction and detection

Total viral RNA was extracted from Vero E6 cells or the tissues homogenates of nasal

and lung using the RNeasy Mini Kit (Qiagen, Hilden, Germany), and viral RNA loads were determined by a SARS-CoV-2 RNA detection kit (Shenzhen Puruikang Biotech, China). The viral RNA loads for the target SARS-CoV-2 N gene was normalized to the standard curve obtained by using a plasmid containing the full-length cDNA of the SARS-CoV-2 N gene. The reactions were performed with CFX96 system (BIO-RAD, America) according to following protocol: 50 °C for 20 min for reverse transcription, followed by 95 °C for 3 min and then 45 cycles of 95 °C for 5 s, 56 °C for 45 s.

TMPyP4 pharmacokinetic study in Syrian hamsters

To assess the pharmacokinetic study of TMPyP4 in vivo, six-week-old male Syrian hamsters were purchased from Merial Vital Laboratory Animal Technology Company. The pharmacokinetic study was performed as previously described[12, 13] with some modification. Briefly, after a single intranasal (i.n.) administration (30 mg/kg), 3 animals per sampling time point (0.83, 0.25, 0.5, 1, 2, 4, 6, 8, 10 and 24 h post-dosing) blood samples were collected from humanely euthanized mice, and centrifuged to isolate plasma. Subsequently, the plasma compound concentrations of TMPyP4 were determined as previously described[14, 15]. The plasma concentration-time profile was plotted using GraphPad Prism and the pharmacokinetic parameters were calculated using PKsolver 2.0[16]. Non-compartment analysis was performed to estimate pharmacokinetic parameters, including the area under the plasma concentration-time curve ($AUC_{0\text{-}t}$ and $AUC_{0\text{-}inf\_obs}$) and apparent terminal half-life ($T_{1/2}$), mean residence time ($MRT_{0\text{-}inf\_obs}$), apparent volume of distribution during terminal phase after non-intravenous administration ($Vz/F_{\_obs}$) and apparent total clearance of the drug from plasma ($CL/F_{\_obs}$), peak plasma concentration (C_{max}), and the time to attain C_{max} (T_{max}).

In vivo toxicity studies of TMPyP4

To assess the toxicity of TMPyP4 in vivo, six-week-old male Syrian hamsters were purchased from Merial Vital Laboratory Animal Technology Company and randomly divided into indicated groups before TMPyP4 or vehicle treatment. TMPyP4 were dissolved in saline. The hamsters were intranasal administration 30 mg/kg TMPyP4 once daily for one week (n = 5). All animals were monitored twice a day for at least 7

days for toxic signs, including body weight loss, food intake reduction and abnormal behaviors. At the end of the experiment, samples of liver, lung and kidney were collected. All tissues were fixed with paraformaldehyde (4%) before dehydration and embedding in paraffin. Paraffin sections were applied for hematoxylin and eosin staining (H&E).

In vivo antiviral studies of TMPyP4

The in vivo antiviral studies were carried out in an ABSL3 facility in Changchun Veterinary Research Institute, Chinese Academy of Agricultural Sciences. All animal studies were performed in strict accordance with the guidelines set by the Chinese Regulations of Laboratory Animals and Laboratory Animal-Requirements of Environment and Housing Facilities. All animals used in this study were chosen randomly. We performed two studies to evaluate the in vivo efficacy of TMPyP4 as antiviral in SARS-CoV-2 infection. First, we use a model of Syrian hamsters; and second, we used a model of human angiotensin-converting enzyme 2 (hACE2)-KI/IOZ C57BL/6 mouse.

The six-week-old male hamsters were intranasal (i.n.) administration TMPyP4 (n = 10) or saline (n = 5) 1 h prior to infection. Then the hamsters were anesthetized and infected intranasally with 10^5 $TCID_{50}$ of SARS-CoV-2. For the low challenge dose group, 15 mg/kg TMPyP4 was given i.n. once daily (n = 5). For the high challenge dose group, 30 mg/kg TMPyP4 was given i.n. once daily (n = 5). The conditions of hamsters including body weight were monitored daily until sacrifice. 3 days post infection (dpi), the hamsters were humanely euthanized. The nasal wash, nasal tissues and lung tissues were harvested for viral titration and pathological examination. Briefly, the nasal tissues and lung tissues were weighed, and 1 gram of each tissue was placed into 1 mL of PBS containing 100 U/mL penicillin, making 10% weight/volume homogenates. The tissue samples were homogenized by Tissue Lyser (QIAGEN, Germany) and centrifuged at 8,000 rpm. The supernatants were then collected and inoculated into Vero E6 cells, and viral RNA copy numbers and viral titration were determined by qRT-PCR and $TCID_{50}$, respectively. Meanwhile, the lungs were fixed in formalin, and the fixed tissues were embedded in paraffin and stained with H&E

for pathological examination. For investigating the antiviral effect of TMPyP4 therapeutic treatment in vivo, the hamsters were pre-infected with the virus (10^5 $TCID_{50}$) for 24 h, and then 30 mg/kg TMPyP4 was given i.n. once daily (n = 5).

The six-week-old male hamsters were administration saline (n = 5), TMPyP4 (n = 5, i.n., 15 mg/kg), TMPyP2 (n = 5, i.n., 15 mg/kg), remdesivir (n = 5, intraperitoneal, i.p., 15 mg/kg) and combination of TMPyP4 (n = 5, i.n., 15 mg/kg) and remdesivir (n = 5, intraperitoneal, i.p., 15 mg/kg)) 1 h prior to infection. Then the hamsters were anesthetized and infected intranasally with 10^5 $TCID_{50}$ of SARS-CoV-2. The conditions of hamsters including body weight were monitored daily until sacrifice. 3 dpi, the hamsters were humanely euthanized. The nasal wash, nasal tissues and lung tissues were harvested for viral titration and pathological examination.

The six-week-old female hACE2-KI/IOZ C57BL/6 mice were purchased from Beijing HFK BIOSCIENCE. Co., Ltd. The mice were i.n. administration TMPyP4 (n = 10) or saline (n = 5) 1 h prior to infection. Then the mice were anesthetized and infected intranasally with 10^5 $TCID_{50}$ of SARS-CoV-2. For the low challenge dose group, 15 mg/kg TMPyP4 was given i.n. once daily (n = 5). For the high challenge dose group, 30 mg/kg TMPyP4 was given i.n. once daily (n = 5). The conditions of mice including body weight were monitored daily until sacrifice. 3 dpi, mice were humanely euthanized. The nasal tissues and lung tissues of mice were harvested and weighed for viral titration, and 0.2 gram of each tissue was placed into 1 mL of PBS for homogenates.

Lung histological analysis

Lung histological analysis was examined by an experienced pathologist. H&E stained sections of lung from hamsters and mice were examined by implementing a semi quantitative, 5 point grading scheme (0-within normal limits, 1-mild, 2-moderate, 3-marked, 4-severe) that took into account four different histopathological parameters: 1) perivascular inflammation 2) bronchial or bronchiolar epithelial degeneration or necrosis 3) bronchial or bronchiolar inflammation and 4) alveolar inflammation.

Reference

1 Varizhuk A, Ischenko D, Tsvetkov V *et al.* The expanding repertoire of G4 DNA structures. *Biochimie* 2017; **135**:54-62.

2 Hon J, Martinek T, Zendulka J, Lexa M. pqsfinder: an exhaustive and imperfection-tolerant search tool for potential quadruplex-forming sequences in R. *Bioinformatics* 2017; **33**:3373-3379.

3 Kikin O, D'Antonio L, Bagga PS. QGRS Mapper: a web-based server for predicting G-quadruplexes in nucleotide sequences. *Nucleic acids research* 2006; **34**:W676-682.

4 Doluca O. G4Catchall: A G-quadruplex prediction approach considering atypical features. *Journal of theoretical biology* 2019; **463**:92-98.

5 Garant JM, Perreault JP, Scott MS. G4RNA screener web server: User focused interface for RNA G-quadruplex prediction. *Biochimie* 2018; **151**:115-118.

6 Beaudoin JD, Jodoin R, Perreault JP. New scoring system to identify RNA G-quadruplex folding. *Nucleic acids research* 2014; **42**:1209-1223.

7 Bedrat A, Lacroix L, Mergny JL. Re-evaluation of G-quadruplex propensity with G4Hunter. *Nucleic acids research* 2016; **44**:1746-1759.

8 Garant JM, Perreault JP, Scott MS. Motif independent identification of potential RNA G-quadruplexes by G4RNA screener. *Bioinformatics* 2017; **33**:3532-3537.

9 Crooks GE, Hon G, Chandonia JM, Brenner SE. WebLogo: a sequence logo generator. *Genome research* 2004; **14**:1188-1190.

10 Qin G, Tu X, Li H *et al.* Long Noncoding RNA p53-Stabilizing and Activating RNA Promotes p53 Signaling by Inhibiting Heterogeneous Nuclear Ribonucleoprotein K deSUMOylation and Suppresses Hepatocellular Carcinoma. *Hepatology* 2020; **71**:112-129.

11 Luo Q, Zhang L, Wei F *et al.* mTORC1 Negatively Regulates the Replication of Classical Swine Fever Virus Through Autophagy and IRES-Dependent Translation. *iScience* 2018; **3**:87-101.

12 White KM, Rosales R, Yildiz S *et al.* Plitidepsin has potent preclinical efficacy against SARS-CoV-2 by targeting the host protein eEF1A. *Science* 2021; **371**:926-931.

13 Qiao J, Li YS, Zeng R *et al.* SARS-CoV-2 M(pro) inhibitors with antiviral activity in a transgenic mouse model. *Science* 2021; **371**:1374-1378.

14 Shioda N, Yabuki Y, Yamaguchi K *et al.* Targeting G-quadruplex DNA as cognitive function therapy for ATR-X syndrome. *Nature medicine* 2018; **24**:802-813.

15 Rapozzi V, Zorzet S, Zacchigna M, Della Pietra E, Cogoi S, Xodo LE. Anticancer activity of cationic porphyrins in melanoma tumour-bearing mice and mechanistic in vitro studies. *Molecular cancer* 2014; **13**:75.

16 Zhang Y, Huo M, Zhou J, Xie S. PKSolver: An add-in program for pharmacokinetic and pharmacodynamic data analysis in Microsoft Excel. *Computer methods and programs in biomedicine* 2010; **99**:306-314.

Table S1. Characteristics of the 18 putative G-quadruplexes

Putative G-quadruplexes	Positions[a]	Genes	Sequences	cGcC[b]	G4H[c]	G4NN[d]
P1	353-377	ORF1ab-nsp1	GGCUUUGGAGACUCCGUGGAGGAGG	3.6667	0.64	0.0214
P2	644-663	ORF1ab-nsp1	GGUAAUAAAGGAGCUGGUGG	**17**	0.8	**0.7006**
P3	1,574-1,599	ORF1ab-nsp2	GGUGUUGUUGGAGAAGGUUCCGAAGG	**5**	0.6154	0.2025
P4	3,467-3,483	ORF1ab-nsp3	GGAGGAGGUGUUGCAGG	**18**	**1**	**0.8704**
P5	8,687-8,709	ORF1ab-nsp4	GGAUACAAGGCUAUUGAUGGUGG	**8.5**	0.6522	0.1148
P6	13,385-13,404	ORF1ab-nsp10	GGUAUGUGGAAAGGUUAUGG	**170**	0.85	0.022
P7	24,215-24,234	S	GGUUGGACCUUUGGUGCAGG	3.4	0.6	0.1581
P8	24,268-24,291	S	GGCUUAUAGGUUUAAUGGUAUUGG	**16**	0.625	0.0053
P9	25,197-25,218	S	GGCCAUGGUACAUUUGGCUAGG	2.6667	0.4545	0.0244
P10	28,903-28,917	N	GGCUGGCAAUGGCGG	**5.3333**	0.8667	**0.568**
P11	14-37	-	GGUUGGUUUGUUACCUGGGAAGG	**5.75**	0.7826	**0.5672**
P12	4,886-4,901	-	GGUGGAAUGUGGUAGG	**170**	**1.0625**	**0.8456**
P13	6,011-6,027	-	GGAUAUGGUUGGUUUGG	**160**	**0.9412**	0.0217
P14	10,015-10,030	-	GGUGAUAGAGGUUUGUGGUGGUUGG	**230**	**0.92**	0.2852
P15	13,254-13,281	-	GGAUUUGGAUGAUCUAUGUGGCAACGG	**6**	0.5556	0.0337
P16	15,924-15,941	-	GGAUCUGGGUAAGGAAGG	**22**	**1.1111**	**0.9722**
P17	16,750-16,768	-	GGUUAAGTGGUGGUCUAGG	**17**	0.8421	**0.7926**

P18	29,718-29,739	-	GGCCUCGGUGAAAAUGUGGUGG	3.6	0.5909	0.2217

[a]Positions are based on SARS-CoV-2 Jan. 2020/NC_045512.2 Assembly (wuhCor1). [b]cGcC was implemented to address the issue of competition in between G4 and Watson-Crick based structures, and the threshold is 4.5. [c]G4H attributes an increasing positive score to each contiguous G and a negative counterpart for contiguous C, the sequence is then scored by the average of the values. The threshold is 0.9. [d]This artificial neural network, G4NN, evaluates the similarity of a given sequence to known G-quadruplexes and reports it as a score between 0 and 1. The threshold is 0.5. cGcC, the consecutive G over consecutive C ratio; G4H, G4Hunter; G4NN, G4 neural network; N, nucleocapsid protein; nsp, non-structural protein; ORF, open reading frame; S, spike glycoprotein; -, putative G-quadruplexes locate in negative-sense strands of SARS-CoV-2; Boldface type indicates that the scores of putative G-quadruplexes are above the threshold.

Table S2. The stability and thermodynamic parameters of 14 putative G-quadruplexes

**Putative G-quadruplexes**	**G4-formation**	T_M	ΔG°_{25} **(kcal/mol)**	ΔH° **(kcal/mol)**	$T\Delta S^\circ$ **(kcal/mol)**
P2	**Yes**	45	-1.8 ± 0.07	-19.8 ± 0.9	-18.0 ± 0.8
P3	**Yes**	50	-2.1 ± 0.1	-20.5 ± 1.3	-18.46 ± 1.1
P4	**Yes**	63.2	-3.1 ± 0.20	-28.0 ±1.3	-24.9 ± 1.2
P5	No	-	-	-	-
P6	**Yes**	41.2	-1.9 ± 0.08	-20.2 ± 1.1	-18.3 ± 0.9
P8	No	-	-	-	-
P10	**Yes**	57.7	-2.6 ± 0.1	-26.7 ± 1.0	-25.1 ± 1.1
P11	No	-	-	-	-
P12	No	-	-	-	-
P13	**Yes**	55.1	-2.3 ± 0.12	-22.5 ± 1.1	-20.2 ± 0.7
P14	**Yes**	56.3	-2.7 ± 0.09	-28.5 ± 1.3	-25.8 ± 1.2
P15	No	-	-	-	-
P16	No	-	-	-	-
P17	No	-	-	-	-

ΔH°, the enthalpy change; ΔG°, the free energy change; $\Delta S^\circ$the entropy change; T_M, melting temperature.

Table S3. Oligonucleotides and primers in this study.

Name	Sequences (5'→3')	Usage
**Oligonucleotides**		
P2 -WT	GGUAAUAAAGGAGCUGGUGG	
P2 -MUT	GAUAAUAAAGAAGCUGAUGA	
P3-WT	GGUGUUGUUGGAGAAGGUUCCGAAGG	
P3-MUT	GAUGUUGUUGAAGAAGAUUCCGAAGA	
P4-WT	GGAGGAGGUGUUGCAGG	
P4-MUT	GAAGAAGAUGUUGCAGA	
P5-WT	GGAUACAAGGCUAUUGAUGGUGG	
P5-MUT	GAAUACAAGACUAUUGAUGAUGA	
P6-WT	GGUAUGUGGAAAGGUUAUGG	
P6-MUT	GAUAUGUGAAAAGAUUAUGA	
P8-WT	GGCUUAUAGGUUUAAUGGUAUUGG	For spectroscopy fluorescence assays, CD measurements and NMR etc.
P8-MUT	GACUUAUAGAUUUAAUGAUAUUGA	
P10-WT	GGCUGGCAAUGGCGG	
P10-MUT	GACUGACAAUGACGA	
P11-WT	GGUUGGUUUGUUACCUGGGAAGG	
P11-MUT	GAUUGAUUUGUUACCUGAGAAGA	
P12-WT	GGUGGAAUGUGGUAGG	
P12-MUT	GAUGAAAUGUGAUAGA	
P13-WT	GGAUAUGGUUGGUUUGG	
P13-MUT	GAAUAUGAUUGAUUUGA	
P14-WT	GGUGAUAGAGGUUUGUGGUGGUUGG	
P14-MUT	GAUGAUAGAGAUUUGUGAUGGUUGA	

P15-WT	GGAUUUGGAUGAUCUAUGUGGCAACGG	
P15-MUT	GAAUUUGAAUGAUCUAUGUGACAACGA	
P16-WT	GGAUCUGGGUAAGGAAGG	
P16-MUT	GAAUCUGAGUAAGAAAGA	
P17-WT	GGUUAAGTGGUGGUCUAGG	
P17-MUT	GAUUAAGTGAUGAUCUAGA	
**Probes**		
1	TGATTTTGGGGTCCATTATC	For FISH assays
2	AACGTAATGCGGGGTGCATT	
3	AGTTGAATCTGAGGGTCCAC	
4	GTTCTCCATTCTGGTTACTG	
5	CGACGTTGTTTTGATCGCGC	
6	AGACGCAGTATTATTGGGTA	
7	TGTTGAGTGAGAGCGGTGAA	
8	GGGAATTTAAGGTCTTCCTT	
9	AATTGGAACGCCTTGTCCTC	
10	CATCTGGACTGCTATTGGTG	
11	CTTCGGTAGTAGCCAATTTG	
12	ACCACGAATTCGTCTGGTAG	
13	GATCTTTCATTTTACCGTCA	
14	TTTGTTAGCACCATAGGGAA	
15	CAGTTGCAACCCATATGATG	
16	GATTGCAGCATTGTTAGCAG	
17	TAGAAGCCTTTTGGCAATGT	
18	ACGAGAAGAGGCTTGACTGC	

19	TGTTGCGACTACGTGATGAG	
20	GCCTGGAGTTGAATTTCTTG	
21	CAGGAGAAGTTCCCCTACTG	
22	CAGCAAAGCAAGAGCAGCAT	
23	AGCTGGTTCAATCTGTCAAG	
24	TTTACCAGACATTTTGCTCT	
25	TAGTGGCAGTACGTTTTTGC	
26	GCTTGTGTTACATTGTATGC	
27	TTTGTTCTGGACCACGTCTG	
28	TGGTCCCCAAAATTTCCTTG	
29	AGTTCCTTGTCTGATTAGTT	
30	CAATTTGCGGCCAATGTTTG	
31	GAAGCGCTGGGGGCAAATTG	
32	ATGCGCGACATTCCGAAGAA	
33	CGAAGGTGTGACTTCCATGC	
34	CTGTGTAGGTCAACCACGTT	
35	ATTTGGATCTTTGTCATCCA	
36	TTGTATGCGTCAATATGCTT	
37	GGTAAGGCTTGAGTTTCATC	
38	GAAGAGTCACAGTTTGCTGT	
39	ATCATCCAAATCTGCAGCAG	
40	GGATTGTTGCAATTGTTTGG	
LNA-ASOs		
P2	ACCAGCTCCTTTATTAC	For ASO assays
P3	GGAACCTTCTCCAACAA	

P4	CCTGCAACACCTCCTCC	
P6	ATAACCTTTCCACATAC	
P10	ACCGCCATTGCCAGCCA	
P13	CCAAACCAACCATATCC	
P14	CCACCACAAACCTCTAT	
**Fluorescently-labeled oligonucleotides**		
P4-WT	Cy5-GGAGGAGGUGUUGCAGG	For immunofluorescence assays
P4-MUT	Cy5-GAAGAAGAUGUUGCAGA	
**qRT-PCR primers for pro-inflammatory cytokines and chemokines**		
IL6-F	ACTCACCTCTTCAGAACGAATTG	Human *IL6* mRNA quantification
IL6-R	CCATCTTTGGAAGGTTCAGGTTG	
CXCL10-F	CTGAGCCTACAGCAGAGGAAC	Human *CXCL10* mRNA quantification
CXCL10-R	GATGCAGGTACAGCGTACAGT	
CXCL11-F	GCATAGGCCCTGGGGTAAAA	Human *CXCL11* mRNA quantification
CXCL11-R	TTGGACTCCTTTGGGCAGTG	
CXCL3-F	GCCCAAACCGAAGTCATAGC	Human *CXCL3* mRNA quantification
CXCL3-R	CCCTGCAGGAAGTGTCAATG	
IFNB1-F	AGTAGGCGACACTGTTCGTG	Human *IFNB1* mRNA quantification
IFNB1-R	AGCCTCCCATTCAATTGCCA	
ISG15-F	CAGCGAACTCATCTTTGCCAG	Human *ISG15* mRNA quantification
ISG15-R	GACACCTGGAATTCGTTGCC	
Il6-F	GACTGGGGATGTCTGTAGCTC	Mouse *Il6* mRNA quantification
Il6-R	CACCAGCATCAGTCCCAAGA	
Cxcl10-F	ACTCCCCTTTACCCAGTGGA	Mouse *Cxcl10* mRNA quantification
Cxcl10-R	AGGACCATGGCTTGACCATC	

Cxcl11-F	CCACGCTACCTTCTGTGGTT	Mouse *Cxcl11* mRNA quantification
Cxcl11-R	ATGTTCGTGTGCCTCGTGAT	
Cxcl3-F	CAGTGCCTGAACACCCTACC	Mouse *Cxcl3* mRNA quantification
Cxcl3-R	GGACTTGCCGCTCTTCAGTA	
Ifnb1-F	CTCCAGCACTGGGTGGAATG	Mouse *Ifnb1* mRNA quantification
Ifnb1-R	AGTTGAGGACATCTCCCACG	
Isg15-F	ACGATTTCCTGGTGTCCGTG	Mouse *Isg15* mRNA quantification
Isg15-R	AGACCCAGACTGGAAAGGGT	
**qRT-PCR primers for RNA replication assays**		
G4 RNA_F	TCTAGGTTTCGTCCGGGTGT	The RNA with G4 site quantification
G4 RNA_R	GCCTCGGTGAAAATGTGGTG	
*GAPDH*_F	CGGAGTCAACGGATTTGGTCGT	*GAPDH* mRNA quantification
*GAPDH*_R	TCTCAGCCTTGACGGTGCCA	

CD, Circular Dichroism; etc., et cetera; F, forward primer; R, reverse primer; qRT-PCR, quantitative real-time polymerase chain reaction.

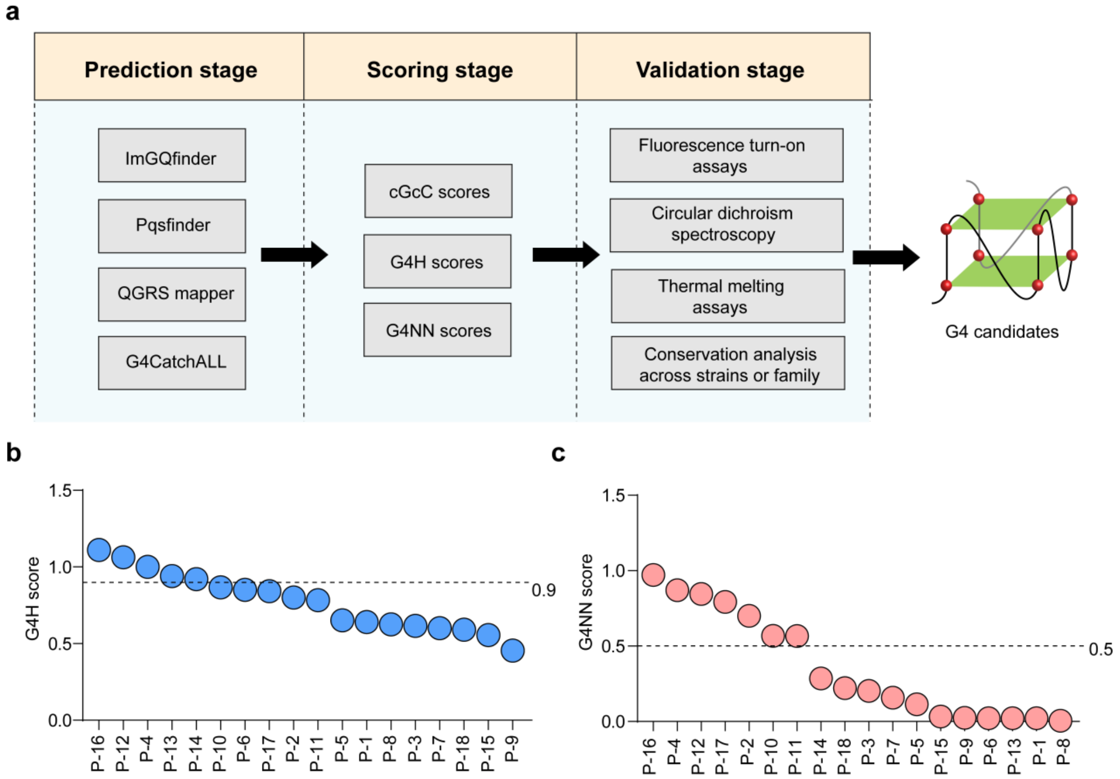
a
Prediction stage
Scoring stage
Validation stage
ImGQfinder
Pqsfinder
QGRS mapper
G4CatchALL
cGcC scores
G4H scores
G4NN scores
Fluorescence turn-on assays
Circular dichroism spectroscopy
Thermal melting assays
Conservation analysis across strains or family
G4 candidates
b
G4H score
1.5
1.0
0.5
0.0
0.9
P-16
P-12
P-4
P-13
P-14
P-10
P-6
P-17
P-2
P-11
P-5
P-1
P-8
P-3
P-7
P-18
P-15
P-9
c
G4NN score
1.5
1.0
0.5
0.0
0.5
P-16
P-4
P-12
P-17
P-2
P-10
P-11
P-14
P-18
P-3
P-7
P-5
P-15
P-9
P-6
P-13
P-1
P-8

Fig. S1 Identification and selection of G-quadruplexes (G4s) in SARS-CoV-2 genome. a Schematic summary of the bioinformatic workflow conducted for PQSs identification and selection in SARS-CoV-2 genome. **b** The G4H score of PQSs in the SARS-CoV-2 genome. G4H, G4hunter. **c** The G4NN score of PQSs in the SARS-CoV-2 genome. G4NN, G4 neural network.

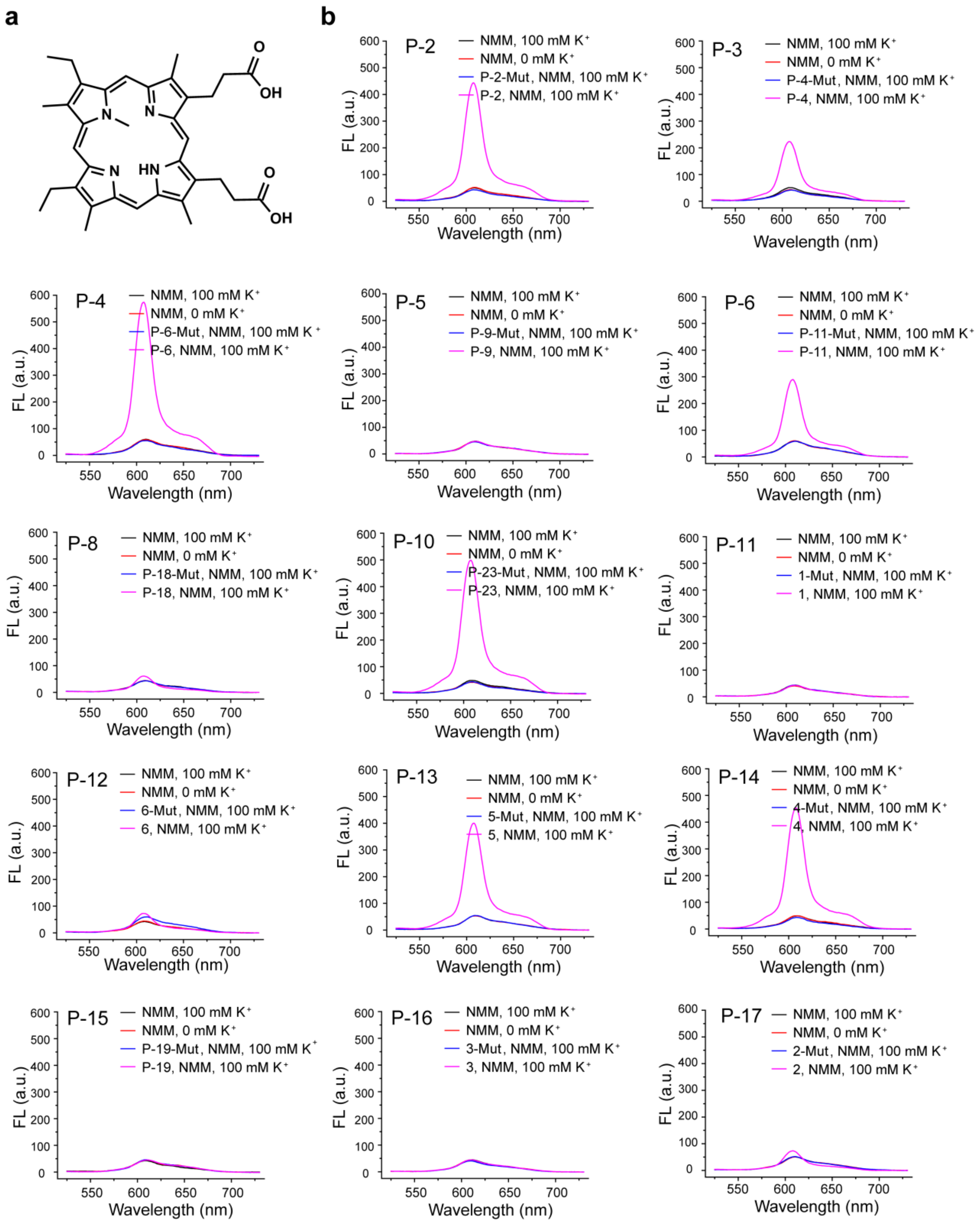

a
O
OH
N
N
N
HN
O
OH
b
P-2
NMM, 100 mM K+
NMM, 0 mM K+
P-2-Mut, NMM, 100 mM K+
P-2, NMM, 100 mM K+
P-3
NMM, 100 mM K+
NMM, 0 mM K+
P-4-Mut, NMM, 100 mM K+
P-4, NMM, 100 mM K+
P-4
NMM, 100 mM K+
NMM, 0 mM K+
P-6-Mut, NMM, 100 mM K+
P-6, NMM, 100 mM K+
P-5
NMM, 100 mM K+
NMM, 0 mM K+
P-9-Mut, NMM, 100 mM K+
P-9, NMM, 100 mM K+
P-6
NMM, 100 mM K+
NMM, 0 mM K+
P-11-Mut, NMM, 100 mM K+
P-11, NMM, 100 mM K+
P-8
NMM, 100 mM K+
NMM, 0 mM K+
P-18-Mut, NMM, 100 mM K+
P-18, NMM, 100 mM K+
P-10
NMM, 100 mM K+
NMM, 0 mM K+
P-23-Mut, NMM, 100 mM K+
P-23, NMM, 100 mM K+
P-11
NMM, 100 mM K+
NMM, 0 mM K+
1-Mut, NMM, 100 mM K+
1, NMM, 100 mM K+
P-12
NMM, 100 mM K+
NMM, 0 mM K+
6-Mut, NMM, 100 mM K+
6, NMM, 100 mM K+
P-13
NMM, 100 mM K+
NMM, 0 mM K+
5-Mut, NMM, 100 mM K+
5, NMM, 100 mM K+
P-14
NMM, 100 mM K+
NMM, 0 mM K+
4-Mut, NMM, 100 mM K+
4, NMM, 100 mM K+
P-15
NMM, 100 mM K+
NMM, 0 mM K+
P-19-Mut, NMM, 100 mM K+
P-19, NMM, 100 mM K+
P-16
NMM, 100 mM K+
NMM, 0 mM K+
3-Mut, NMM, 100 mM K+
3, NMM, 100 mM K+
P-17
NMM, 100 mM K+
NMM, 0 mM K+
2-Mut, NMM, 100 mM K+
2, NMM, 100 mM K+
FL (a.u.)
Wavelength (nm)
0
100
200
300
400
500
600
550
600
650
700

Fig. S2 Screening the PQSs in SARS-CoV-2 genome by fluorescence turn-on assays. a Chemical structures of G4 specific small molecule N-methyl mesoporphyrin IX (NMM). **b** Fluorescence turn-on assays of NMM in the absence or presence of all PQSs (P2, P3, P4, P5, P6, P8, P10, P11, P12, P13, P14, P15, P16 and P17) and their G/A mutants under indicated conditions. PQSs, putative G4-forming sequences.

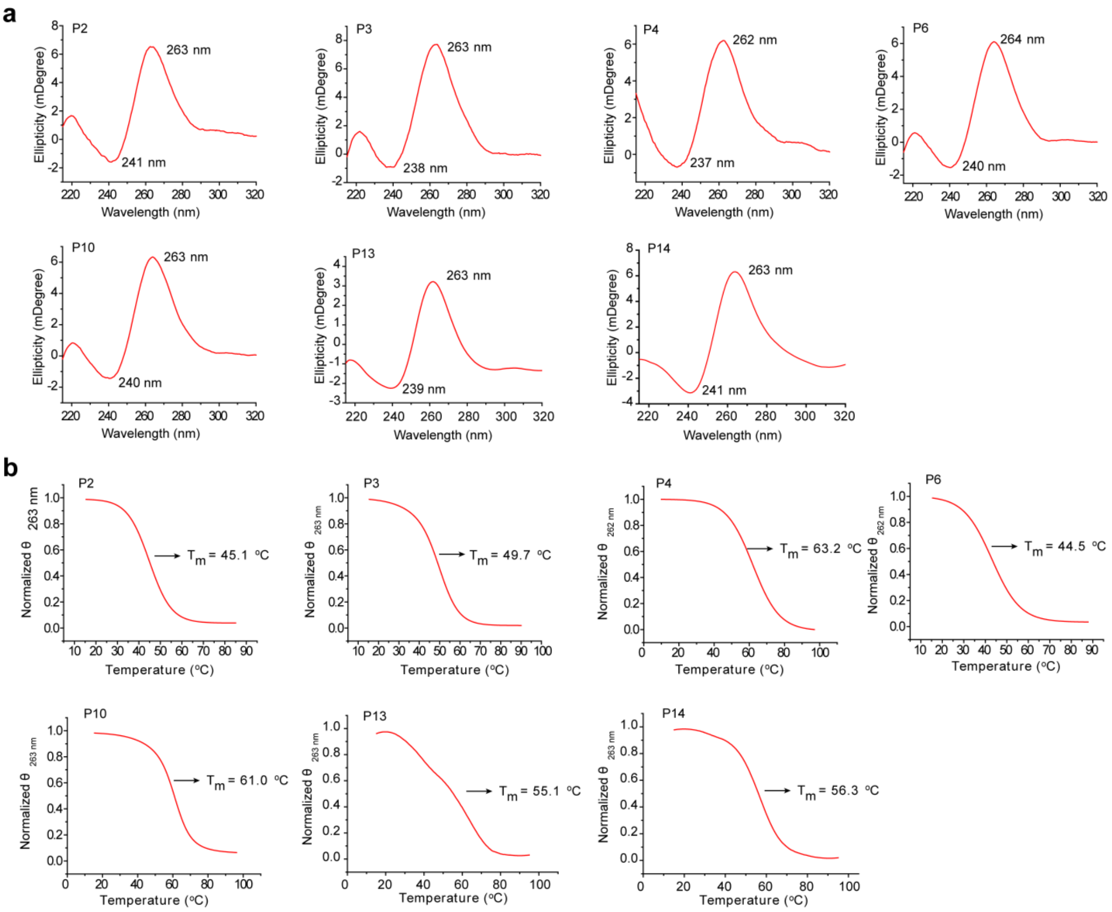
a
P2
263 nm
241 nm
P3
263 nm
238 nm
P4
262 nm
237 nm
P6
264 nm
240 nm
P10
263 nm
240 nm
P13
263 nm
239 nm
P14
263 nm
241 nm
Ellipticity (mDegree)
Wavelength (nm)
b
P2
Tm = 45.1 °C
P3
Tm = 49.7 °C
P4
Tm = 63.2 °C
P6
Tm = 44.5 °C
P10
Tm = 61.0 °C
P13
Tm = 55.1 °C
P14
Tm = 56.3 °C
Normalized θ 263 nm
Normalized θ 262 nm
Temperature (°C)

Fig. S3 The structure and stability of PQS candidates. a CD spectroscopy of PQS candidates (P2, P3, P4, P6, P10, P13, P14) and their G/A mutants. **b** CD thermal melting curves of PQS candidates and their G/A mutants. CD, circular dichroism.

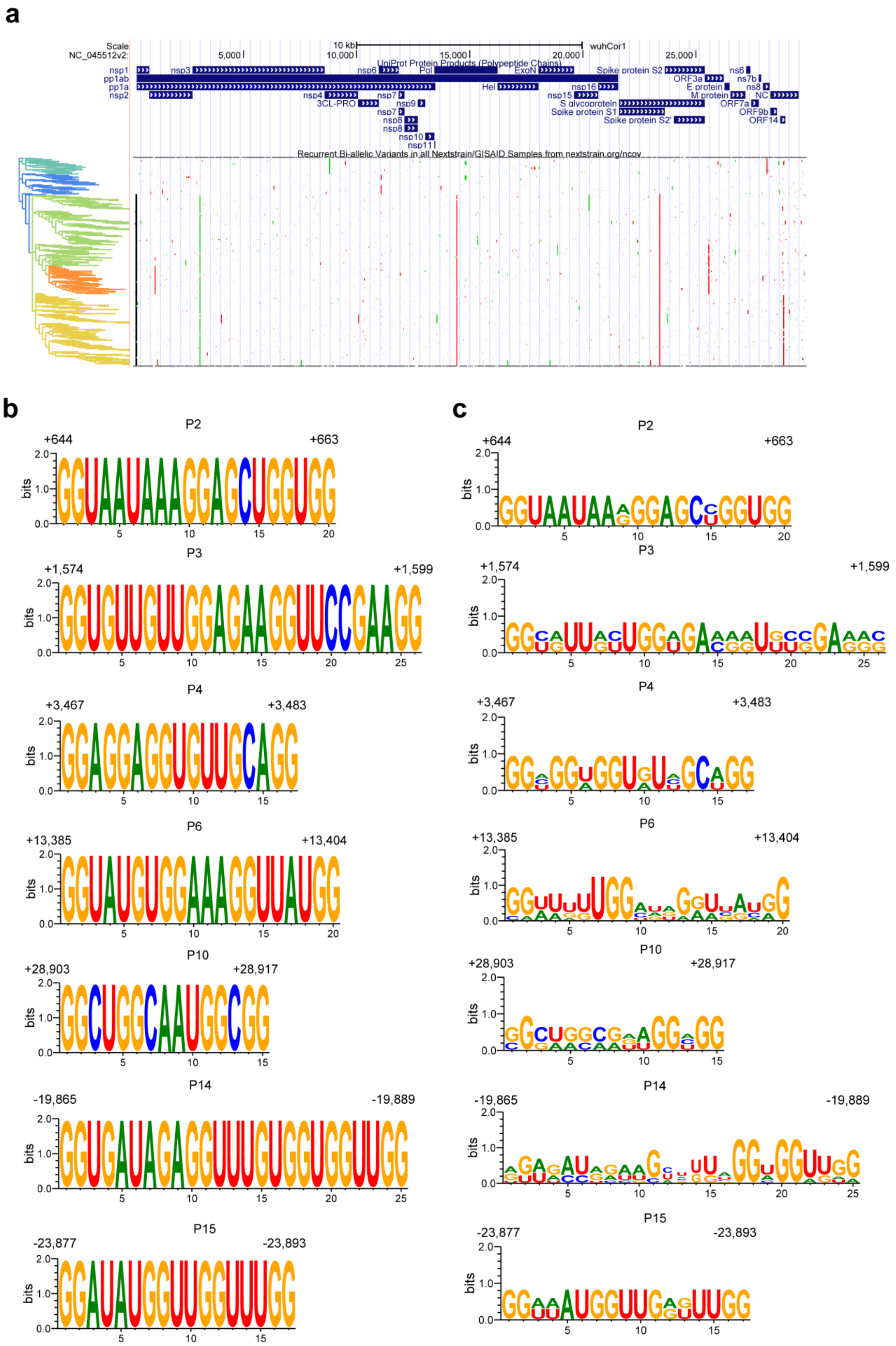
a
b
c
P2
+644
+663
P3
+1,574
+1,599
P4
+3,467
+3,483
P6
+13,385
+13,404
P10
+28,903
+28,917
P14
-19,865
-19,889
P15
-23,877
-23,893
bits
Recurrent Bi-allelic Variants in all Nextstrain/GISAID Samples from nextstrain.org/ncov
UniProt Protein Products (Polypeptide Chains)

Fig. S4 The analysis of the conservation of PQS candidates. a Recurrent Bi-allelic Variants in all Nextstrain/GISAID Samples from nextstrain.org/ncov. **b** The analysis of conservation of the PQS candidates across strains by using the WebLogo 3 software program. **c** The analysis of conservation of the PQS candidates throughout the human coronavirus family by using the WebLogo 3 software program.

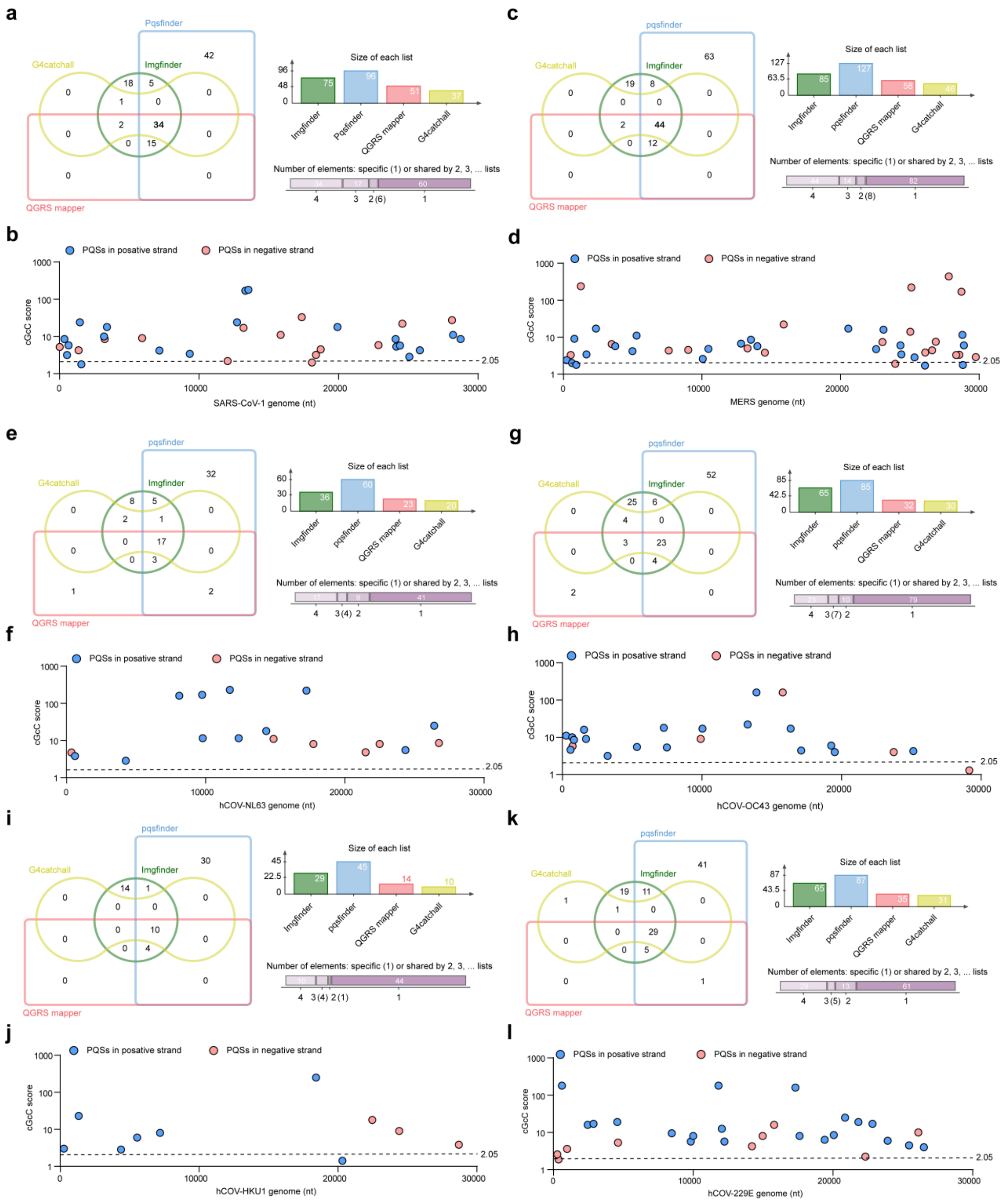

a
Pqsfinder
G4catchall
Imgfinder
QGRS mapper
Size of each list
Number of elements: specific (1) or shared by 2, 3, ... lists
b
PQSs in posative strand
PQSs in negative strand
cGcC score
SARS-CoV-1 genome (nt)
c
d
MERS genome (nt)
e
f
hCOV-NL63 genome (nt)
g
h
hCOV-OC43 genome (nt)
i
j
hCOV-HKU1 genome (nt)
k
l
hCOV-229E genome (nt)

Fig. S5 Visualization of the PQSs in six human coronaviruses. a Edwards' Venn diagram showing the overlap of the putative G4-forming sequences (PQSs) predicted by four independent software (G4catchall, QGRS mapper, Pqsfinder and ImGQfinder) in SARS-CoV-1 genome. Charts showing the list size and intersection size repartition located underneath the diagram. **b** The cGcC score and location of PQSs in the MERS-CoV genome. The cG/cC score of PQSs is calculated by using G4RNA screener. **c** Edwards' Venn diagram showing the overlap of the putative G4-forming sequences (PQSs) predicted by four independent software in MERS-CoV genome. **d** The cGcC score and location of PQSs in the MERS-CoV genome. **e** Edwards' Venn diagram showing the overlap of the putative G4-forming sequences (PQSs) predicted by four independent software in HCoV-NL63 genome. **f** The cGcC score and location of PQSs in the HCoV-NL63 genome. **g** Edwards' Venn diagram showing the overlap of the putative G4-forming sequences (PQSs) predicted by four independent software in HCoV-OC43 genome. **h** The cGcC score and location of PQSs in the HCoV-OC43 genome. **i** Edwards' Venn diagram showing the overlap of the putative G4-forming sequences (PQSs) predicted by four independent software in HCoV-HKU1 genome. **j** The cGcC score and location of PQSs in the HCoV-HKU1 genome. **k** Edwards' Venn diagram showing the overlap of the putative G4-forming sequences (PQSs) predicted by four independent software in HCoV-229E genome. **l** The cGcC score and location of PQSs in the HCoV-229E genome.

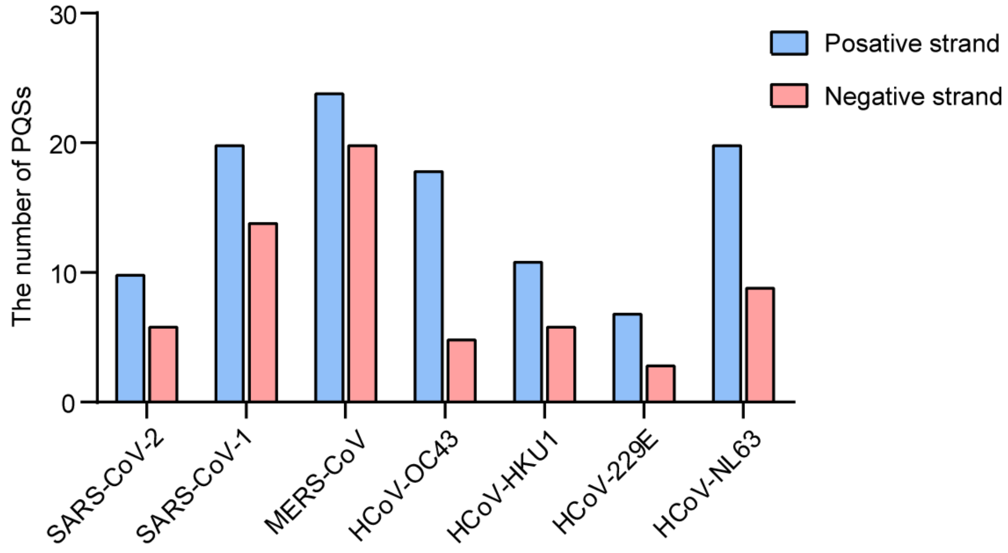
30
20
10
0
The number of PQSs
Posative strand
Negative strand
SARS-CoV-2
SARS-CoV-1
MERS-CoV
HCoV-OC43
HCoV-HKU1
HCoV-229E
HCoV-NL63

Fig. S6 The number of PQSs in seven human coronaviruses. The blue column represents the G4 numbers in positive strand, and the red shows the numbers in negative strand.

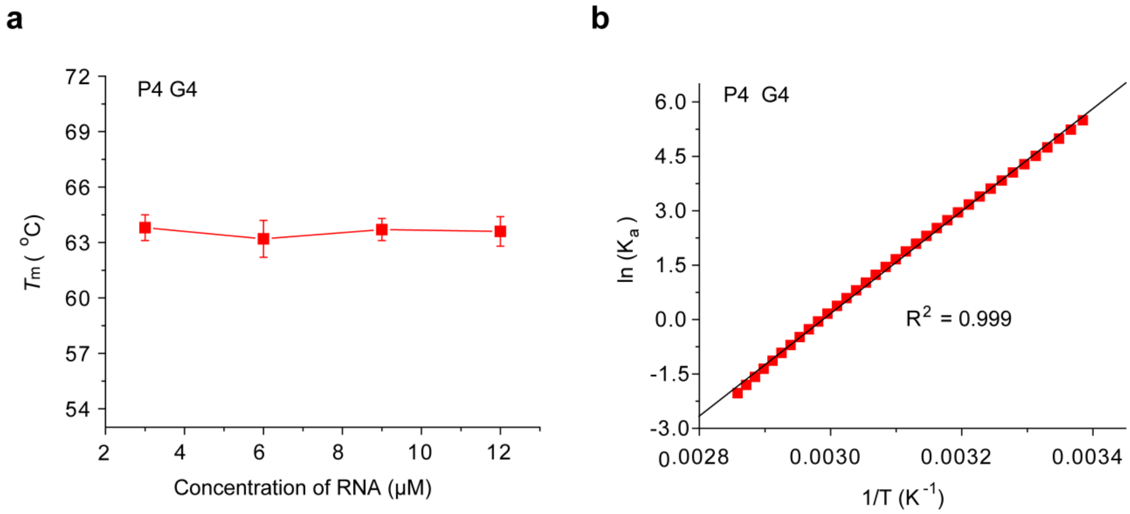
a
P4 G4
72
69
66
63
60
57
54
Tm (°C)
2
4
6
8
10
12
Concentration of RNA (μM)
b
P4 G4
6.0
4.5
3.0
1.5
0.0
-1.5
-3.0
ln (Ka)
R2 = 0.999
0.0028
0.0030
0.0032
0.0034
1/T (K-1)

Fig. S7 The formation of P4 G4 in vitro. a Plots of melting temperature (T_m) versus concentration of P4. Melting assays were carried out in 10 mM Tris-HCl, 100 mM KCl, pH = 7.2 buffer. **b** Determination of $\Delta G°25$, $\Delta H°$ and $\Delta S°$ for P4 G4 formation. The thermodynamic parameters were estimated from the melting measurements. Experiments were carried out in 10 mM Tris-HCl, 100 mM KCl, pH = 7.2 buffer.

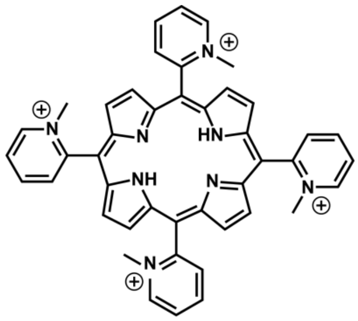

TMPyP2

Fig. S8 Chemical structures of TMPyP2.

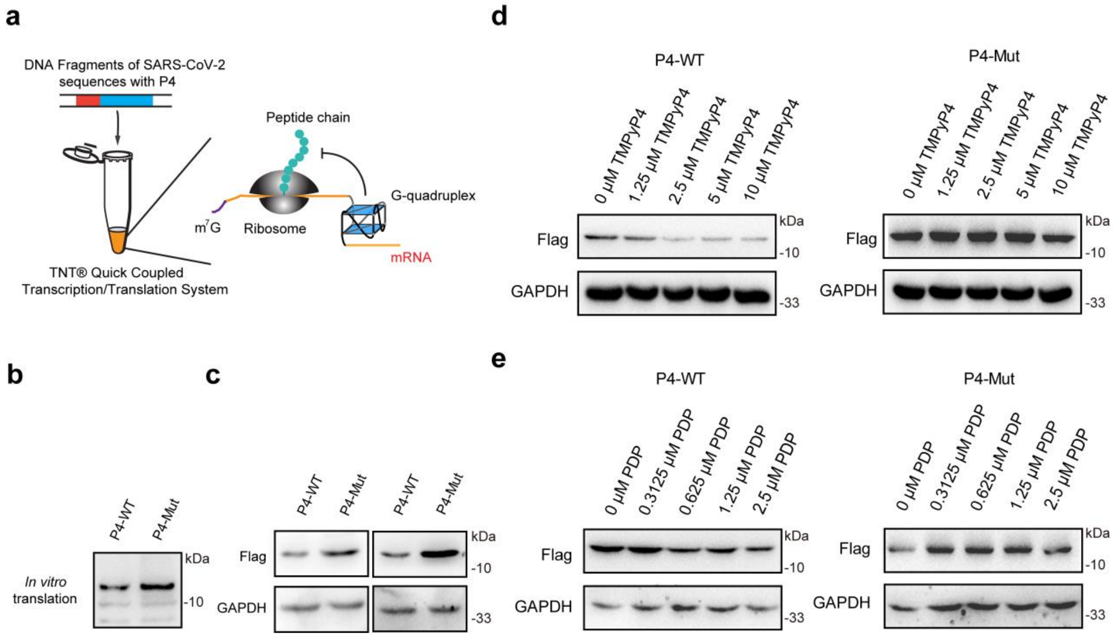
a
DNA Fragments of SARS-CoV-2 sequences with P4
Peptide chain
G-quadruplex
m7G
Ribosome
mRNA
TNT® Quick Coupled Transcription/Translation System
b
P4-WT
P4-Mut
In vitro translation
kDa
-10
c
P4-WT
P4-Mut
P4-WT
P4-Mut
Flag
GAPDH
kDa
-10
-33
d
P4-WT
0 μM TMPyP4
1.25 μM TMPyP4
2.5 μM TMPyP4
5 μM TMPyP4
10 μM TMPyP4
Flag
GAPDH
kDa
-10
-33
P4-Mut
0 μM TMPyP4
1.25 μM TMPyP4
2.5 μM TMPyP4
5 μM TMPyP4
10 μM TMPyP4
Flag
GAPDH
kDa
-10
-33
e
P4-WT
0 μM PDP
0.3125 μM PDP
0.625 μM PDP
1.25 μM PDP
2.5 μM PDP
Flag
GAPDH
kDa
-10
-33
P4-Mut
0 μM PDP
0.3125 μM PDP
0.625 μM PDP
1.25 μM PDP
2.5 μM PDP
Flag
GAPDH
kDa
-10
-33

Fig. S9 The formation of P4 G4 inhibits the protein expression. a Schematic representation of in vitro translation (IVT) assays. **b** G4 formation of P4 inhibits mRNA translation by performing IVT assays. **c** G4 formation of P4 inhibits protein expression in HEK293T and Hela cells. **d** TMPyP4 treatment inhibits the protein expression by targeting P4 G4 in HEK293T cells in a dose-dependent manner, but not P4-Mut. **e** PDP treatment inhibits the protein expression by targeting P4 G4 in HEK293T cells in a dose-dependent manner, but not P4-Mut.

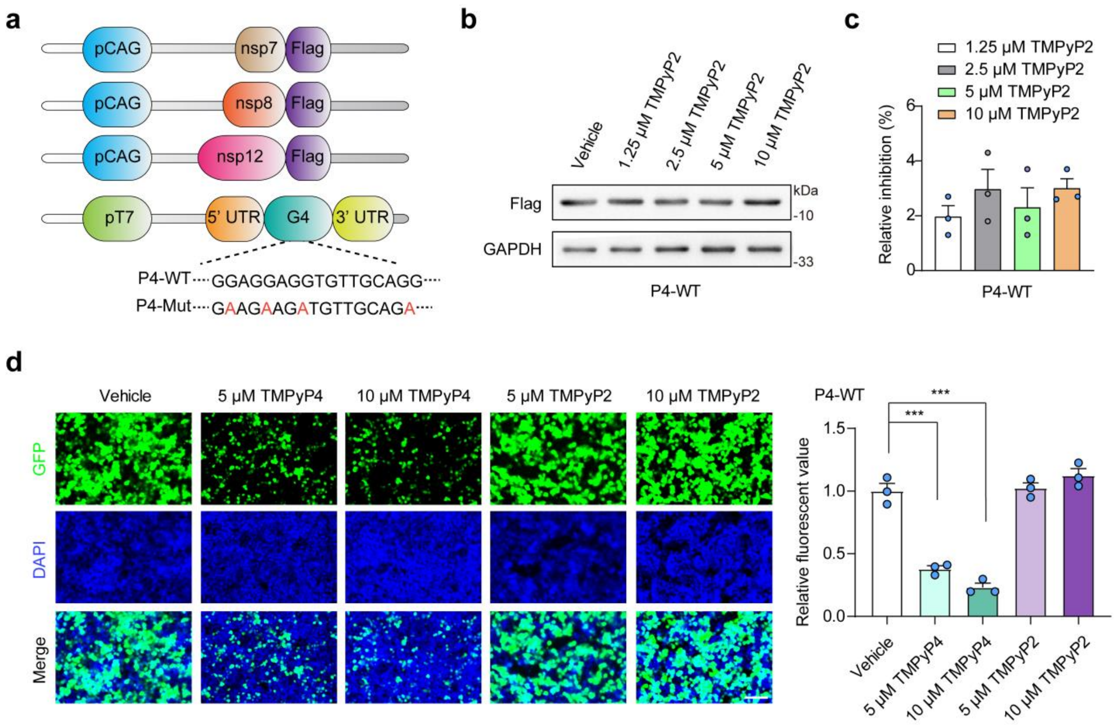
a
pCAG
nsp7
Flag
nsp8
nsp12
pT7
5' UTR
G4
3' UTR
P4-WT····GGAGGAGGTGTTGCAGG····
P4-Mut····GAAGAAGATGTTGCAGA····
b
Vehicle
1.25 μM TMPyP2
2.5 μM TMPyP2
5 μM TMPyP2
10 μM TMPyP2
Flag
GAPDH
kDa
-10
-33
P4-WT
c
1.25 μM TMPyP2
2.5 μM TMPyP2
5 μM TMPyP2
10 μM TMPyP2
Relative inhibition (%)
P4-WT
d
Vehicle
5 μM TMPyP4
10 μM TMPyP4
5 μM TMPyP2
10 μM TMPyP2
GFP
DAPI
Merge
P4-WT
***
Relative fluorescent value

Fig. S10 TMPyP2 treatment does not inhibit the RNA translation and replication. **a** The expression vectors of SARS-CoV-2 RNA-dependent RNA polymerase complexes (nsp7, nsp8 and nsp12). The pGEMT vectors carrying P4 sequences fused to SARS-CoV-2 5'UTR and 3'UTR. **b** TMPyP2 treatment shows no effects on the protein expression of P4-WT in HEK293T cells. **c** TMPyP2 treatment shows no effects on the RNA replication of RNA with P13 G4 in HEK293T cells, detected by qRT-PCR assays. **d** HEK293T cells infected with P4-EGFP lentivirus were treated with or without TMPyP4 or TMPyP2 as indicated. Left: representative confocal images were demonstrated. Scale bars: 100 μm. Right: the relative fluorescent value was measured. Data are shown as mean ± SEM of three independent experiments, two-tailed Student's t test. SEM, standard error of mean. $^{***}P < 0.001$.

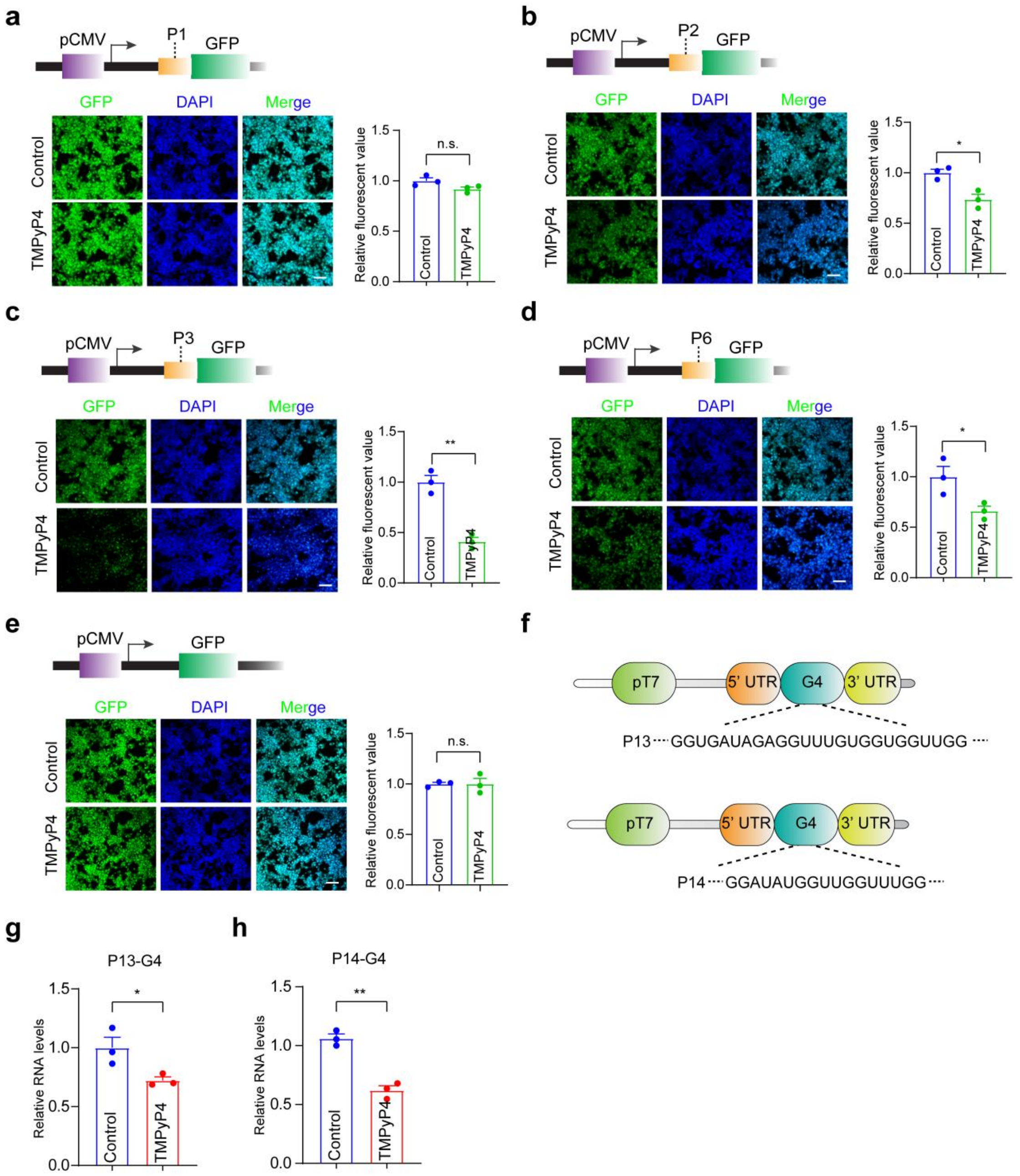
a
pCMV
P1
GFP
GFP
DAPI
Merge
Control
TMPyP4
n.s.
Relative fluorescent value
b
pCMV
P2
GFP
*
c
pCMV
P3
GFP
**
d
pCMV
P6
GFP
*
e
pCMV
GFP
n.s.
f
pT7
5' UTR
G4
3' UTR
P13 ···· GGUGAUAGAGGUUUGUGGUGGUUGG ····
P14 ···· GGAUAUGGUUGGUUUGG ····
g
P13-G4
Relative RNA levels
*
h
P14-G4
**

Fig. S11 TMPyP4 treatment suppresses the RNA translation and replication by targeting the other G4s in SARS-CoV-2. **a-e** HEK293T cells transfected with P1-EGFP (a), P2-EGFP (b), P3-EGFP (c) P6-EGFP (d) and EGFP (e) were treated with or without 10 μM TMPyP4. Confocal fluorescence microphotographs of the same cells with GFP (left), DAPI (middle) or Merge (right) were demonstrated (scale bar = 50 μm). The relative fluorescent value was measured. **f** The pGEMT vectors carrying P13 or P14 sequences fused to SARS-CoV-2 5'UTR and 3'UTR. **g** TMPyP4 treatment inhibited the replication of RNA with P13 G4 in HEK293T cells, detected by qRT-PCR assays. **h** TMPyP4 treatment inhibited the replication of RNA with P14 G4 in HEK293T cells, detected by qRT-PCR assays. Data are shown as mean ± SEM of three independent experiments, two-tailed Student's t test. SEM, standard error of mean. n.s., not significant. $^{*}P < 0.05$, $^{**}P < 0.01$.

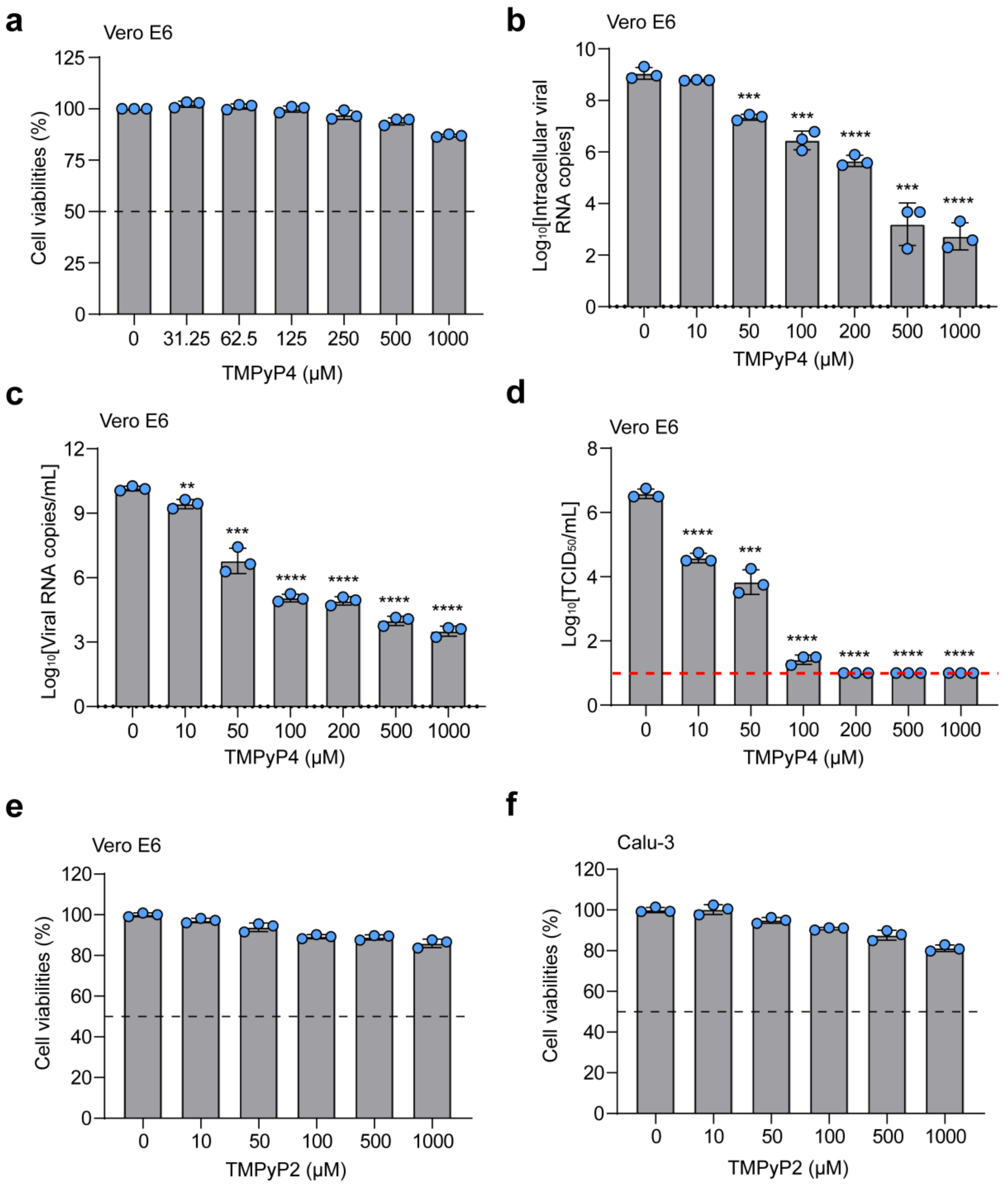
a
Vero E6
Cell viabilities (%)
125
100
75
50
25
0
0
31.25
62.5
125
250
500
1000
TMPyP4 (μM)
b
Vero E6
Log10[Intracellular viral RNA copies]
10
8
6
4
2
0
***
***
****
***
****
0
10
50
100
200
500
1000
TMPyP4 (μM)
c
Vero E6
Log10[Viral RNA copies/mL]
12
9
6
3
0
**
***
****
****
****
****
0
10
50
100
200
500
1000
TMPyP4 (μM)
d
Vero E6
Log10[TCID50/mL]
8
6
4
2
0
****
***
****
****
****
****
0
10
50
100
200
500
1000
TMPyP4 (μM)
e
Vero E6
Cell viabilities (%)
120
100
80
60
40
20
0
0
10
50
100
500
1000
TMPyP2 (μM)
f
Calu-3
Cell viabilities (%)
120
100
80
60
40
20
0
0
10
50
100
500
1000
TMPyP2 (μM)

Fig. S12 The cytotoxicity and antiviral activity of TMPyP4 and TMPyP2 treatment in Vero E6 cells. **a** The cytopathic effect caused by different doses of TMPyP4 treatment for 48 h in Vero E6 cells was quantitatively analyzed by using SRB assays. **b** Vero E6 cells were infected with SARS-CoV-2 (10^2 $TCID_{50}$ virus/mL) and treated with different concentrations of TMPyP4. At 3 dpi, viral RNA copies (per mL) were quantified from cell lysates by qRT-PCR. **c** Vero E6 cells were infected with SARS-CoV-2 (10^2 $TCID_{50}$ virus/mL) and treated with different concentrations of TMPyP4. At 3 day post infection (dpi), viral RNA copies (per mL) were quantified from cell culture supernatants by qRT-PCR. **d** Vero E6 cells were infected with SARS-CoV-2 (10^2 $TCID_{50}$ virus/mL) and treated with different concentrations of TMPyP4. At 3 dpi, viral loads ($log_{10}TCID_{50}$/mL) were quantified by $TCID_{50}$. Lower limit of detection (LLOD) for viral titers is indicated with a red dotted line. **e,f** The cytopathic effect caused by different doses of TMPyP4 treatment for 48 h in Vero E6 (Ee) or Calu-3 (f) cells was quantitatively analyzed by using SRB assays. Data are shown as mean ± SEM of three independent experiments, two-tailed Student's t test. $^{**}P < 0.01$, $^{***}P < 0.001$, $^{****}P < 0.0001$.

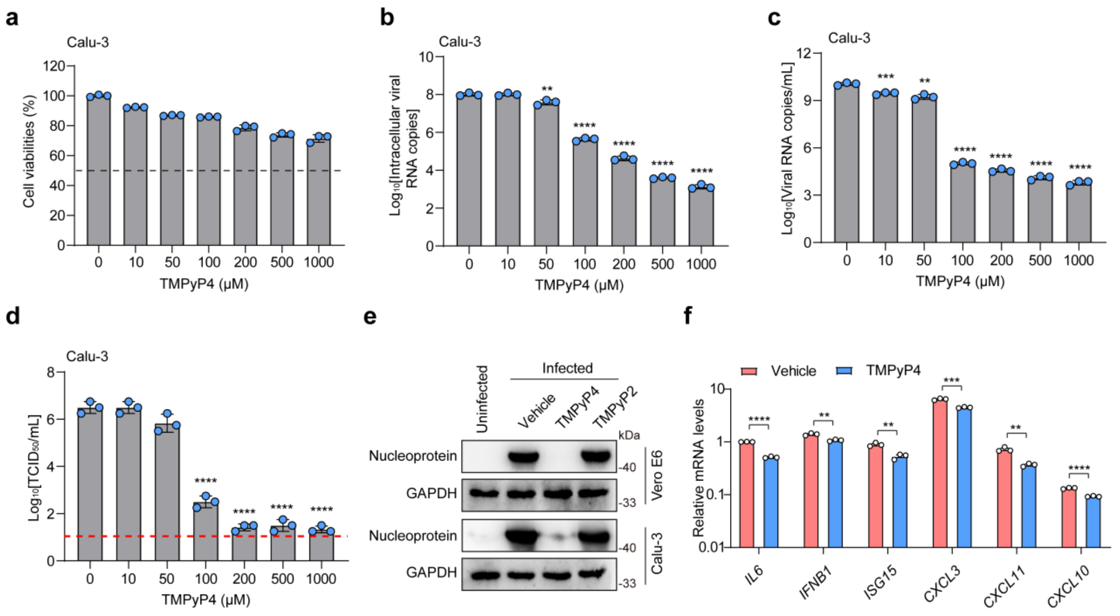
a
Calu-3
Cell viabilities (%)
TMPyP4 (μM)
b
Calu-3
Log10[Intracellular viral RNA copies]
TMPyP4 (μM)
c
Calu-3
Log10[Viral RNA copies/mL]
TMPyP4 (μM)
d
Calu-3
Log10[TCID50/mL]
TMPyP4 (μM)
e
Uninfected
Infected
Vehicle
TMPyP4
TMPyP2
kDa
Nucleoprotein
GAPDH
Vero E6
Calu-3
f
Vehicle
TMPyP4
Relative mRNA levels
IL6
IFNB1
ISG15
CXCL3
CXCL11
CXCL10

Fig. S13 The cytotoxicity and antiviral activity of TMPyP4 in Calu-3 cells. **a** The cytopathic effect caused by different doses of TMPyP4 treatment for 48 h in Calu-3 cells was quantitatively analyzed by using SRB assays. **b** Calu-3 cells were infected with SARS-CoV-2 (10^3 $TCID_{50}$ virus/mL) and treated with different concentrations of TMPyP4. At 3 dpi, viral RNA copies (per mL) were quantified from cell lysates by qRT-PCR. **c** Calu-3 cells were infected with SARS-CoV-2 (10^3 $TCID_{50}$ virus/mL) and treated with different concentrations of TMPyP4. At 3 day post infection (dpi), viral RNA copies (per mL) were quantified from cell culture supernatants by qRT-PCR. **d** Calu-3 cells were infected with SARS-CoV-2 (10^3 $TCID_{50}$ virus/mL) and treated with different concentrations of TMPyP4. At 3 dpi, viral titers ($log_{10}TCID_{50}$/mL) were quantified by $TCID_{50}$. Lower limit of detection (LLOD) for viral titers is indicated with a red dotted line. **e** Vero E6 and Calu-3 cells were infected with SARS-CoV-2 (10^3 $TCID_{50}$ virus/mL) and treated with 100 μM TMPyP4 or 100 μM TMPyP2. The protein expression of viral nucleoprotein was detected by western blotting assays. **f** Calu-3 cells were infected with SARS-CoV-2 (10^3 $TCID_{50}$ virus/mL) and treated with 100 μM TMPyP4. The mRNA levels of chemokine and cytokine were detected by qRT-PCR. Data are shown as mean ± SEM of three independent experiments, two-tailed Student's t test. $^{**}P < 0.01$, $^{***}P < 0.001$, $^{****}P < 0.0001$.

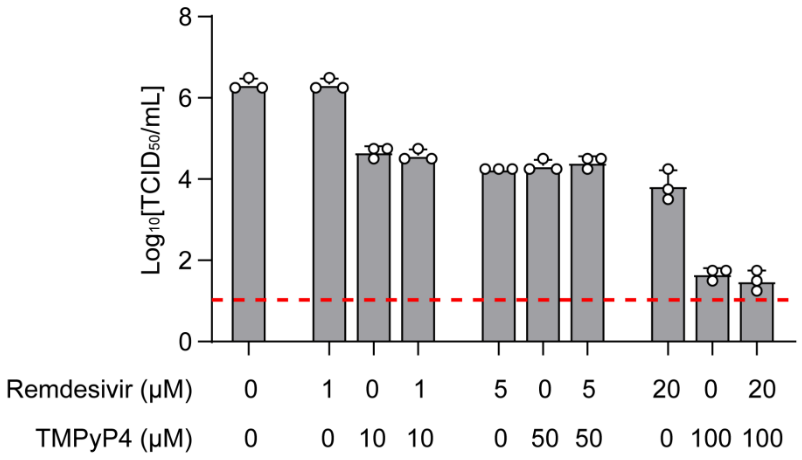

Log10[TCID50/mL]
8
6
4
2
0
Remdesivir (μM) 0 1 0 1 5 0 5 20 0 20
TMPyP4 (μM) 0 0 10 10 0 50 50 0 100 100

Fig. S14 TMPyP4 did not exhibit antiviral synergy with remdesivir. Vero E6 cells were infected with SARS-CoV-2 (10^3 $TCID_{50}$ virus/mL) and treated with different concentrations of TMPyP4 or remdesivir as indicated. At 3 dpi, viral titers ($log_{10}TCID_{50}$/mL) were quantified by $TCID_{50}$. Lower limit of detection for viral titers is indicated with a red dotted line. Data are shown as mean ± SEM of three independent experiments.

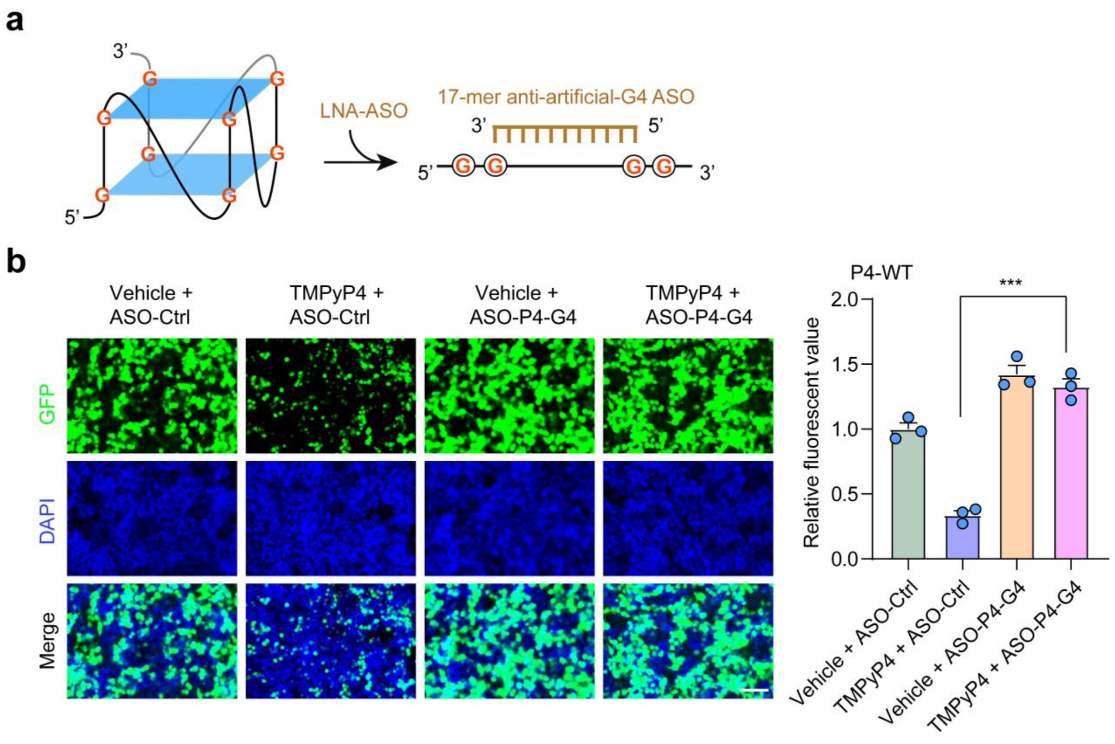
a
3'
5'
G
LNA-ASO
17-mer anti-artificial-G4 ASO
3'
5'
5'
3'
b
Vehicle + ASO-Ctrl
TMPyP4 + ASO-Ctrl
Vehicle + ASO-P4-G4
TMPyP4 + ASO-P4-G4
GFP
DAPI
Merge
P4-WT
***
Relative fluorescent value
2.0
1.5
1.0
0.5
0.0
Vehicle + ASO-Ctrl
TMPyP4 + ASO-Ctrl
Vehicle + ASO-P4-G4
TMPyP4 + ASO-P4-G4

Fig. S15 ASO suppresses the effect of TMPyP4 on P4-EGFP SARS-CoV-2 lentivirus infection. a Schematic representation of the antisense oligonucleotide-based strategy to unfold viral G4 structures. **b** HEK293T cells infected with P4-EGFP lentivirus treated with or without 10 μM TMPyP4 were pre-transfected with the ASOs targeting P4 G4. Left: representative confocal images were demonstrated. Scale bars: 100 μm. Right: the relative fluorescent value was measured. Data are shown as mean ± SEM of three independent experiments, two-tailed Student's t test. $^{***}P < 0.001$.

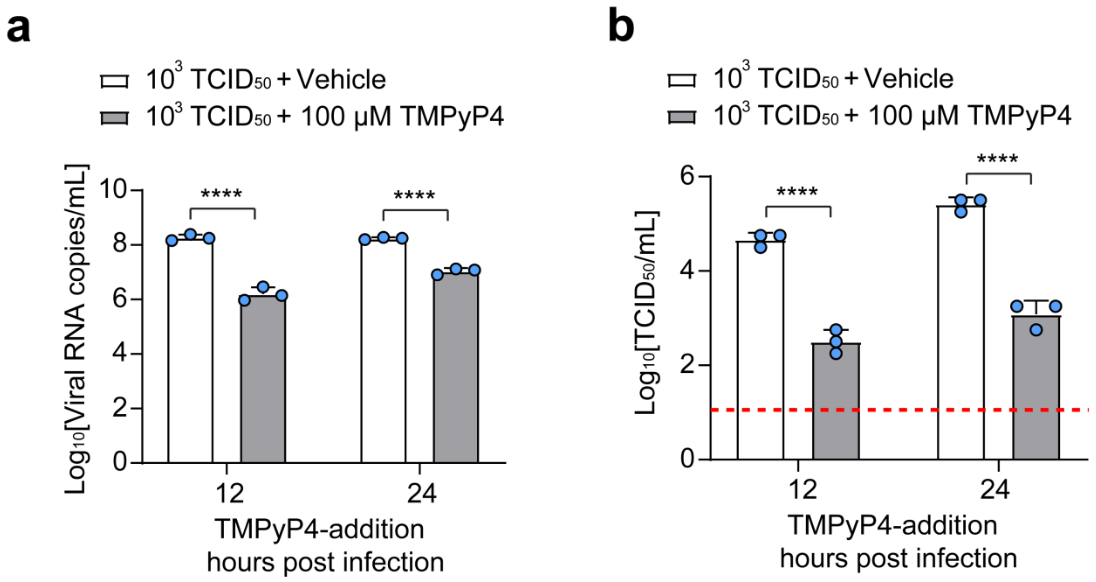
a
10³ TCID50 + Vehicle
10³ TCID50 + 100 μM TMPyP4
Log10[Viral RNA copies/mL]
10
8
6
4
2
0
****
****
12
24
TMPyP4-addition
hours post infection
b
10³ TCID50 + Vehicle
10³ TCID50 + 100 μM TMPyP4
Log10[TCID50/mL]
6
4
2
0
****
****
12
24
TMPyP4-addition
hours post infection

Fig. S16 Therapeutic treatment of TMPyP4 inhibits viral replication in Vero E6 cells. a,b The Vero E6 cells treated with 100 μM TMPyP4 were pre-infected with SARS-CoV-2 (10^3 $TCID_{50}$ virus/mL) for 12 or 24 h. At 48 h post TMPyP4 treatment, viral RNA copies in cell culture supernatants (a) were detected by qRT-PCR. Viral titers (log_{10}TCID50/mL) (b) were quantified by $TCID_{50}$. Lower limit of detection for viral titers is indicated with a red dotted line. Data are shown as mean ± SEM, two-tailed Student's t test. $^{****}P < 0.0001$.

A

Compound	Admin	$t_{1/2}$	T_{max}	C_{max}	AUC_{0-t}	AUC_{0-inf_obs}	$AUMC_{0-inf\_obs}$	MRT_{0-inf_obs}	$Vz/F_{\_obs}$	$Cl/F_{\_obs}$
TMPyP4	i.n. (30 mg/kg)	h	h	μg/mL	μg/mL*h	μg/mL*h	μg/mL*h^2	h	(mg)/(μg/mL)	(mg)/(μg/mL)/h
		6.35763 ± 0.66817	1.00000 ± 0.00000	17.87750 ± 1.23902	155.27837 ± 7.56589	167.31544 ± 11.35503	1519.69406 ± 239.47784	9.04660 ± 0.82337	1.64223 ± 0.08137	0.17985 ± 0.01201

B

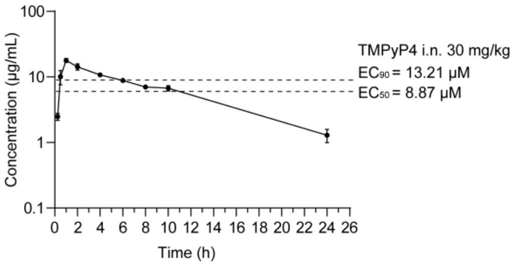

Fig. S17 Primary pharmacokinetic (PK) evaluation of TMPyP4 in hamsters. a Primary PK evaluation of TMPyP4 (i.n. 30 mg/kg) in hamsters (*n* = 3). **b** The mean plasma concentration-time curves of TMPyP4 (i.n. 30 mg/kg).

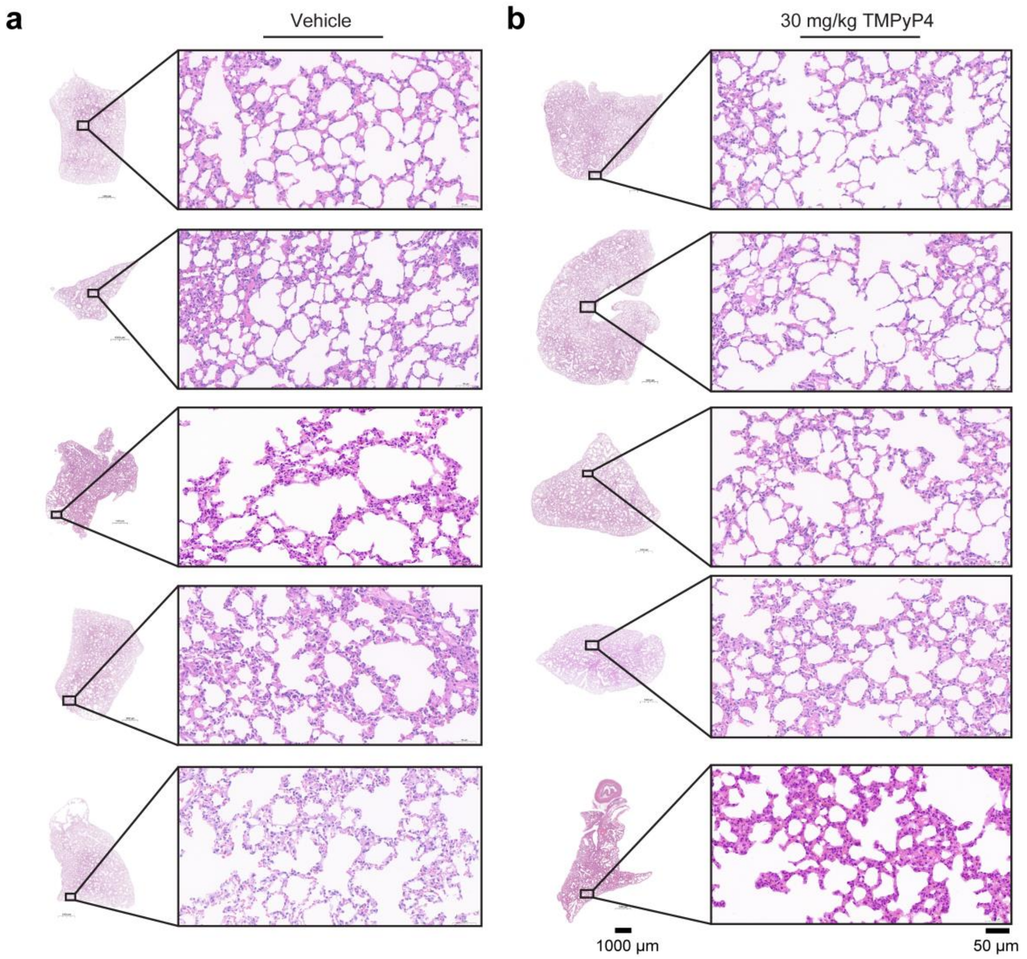
a
Vehicle
b
30 mg/kg TMPyP4
1000 μm
50 μm

Fig. S18 Intranasal administration of TMPyP4 (30 mg/kg) once daily for 7 consecutive days show no noticeable toxicity in the lung tissues of hamsters. a,b The whole lung slide scan images from vehicle (a) and TMPyP4-treated (b) hamsters (*n* = 5).

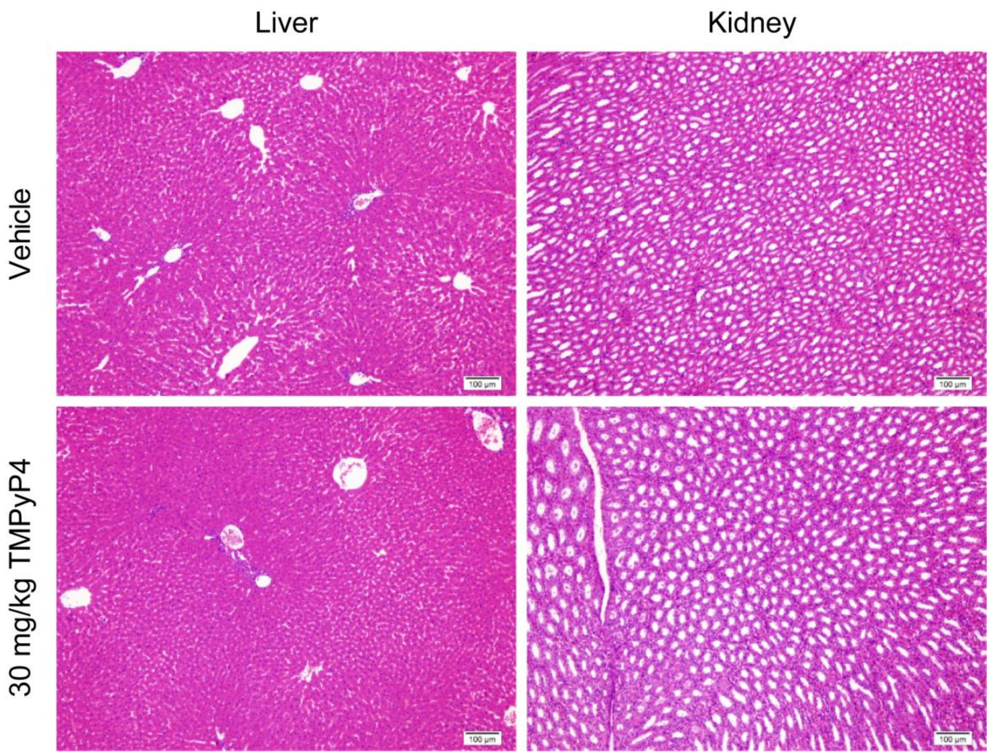
Liver
Kidney
Vehicle
30 mg/kg TMPyP4
100 μm

Fig. S19 Intranasal administration of TMPyP4 (30 mg/kg) once daily for 7 consecutive days show no noticeable toxicity in the liver and kidney tissues of hamsters. Scale bar represents 100 μm.

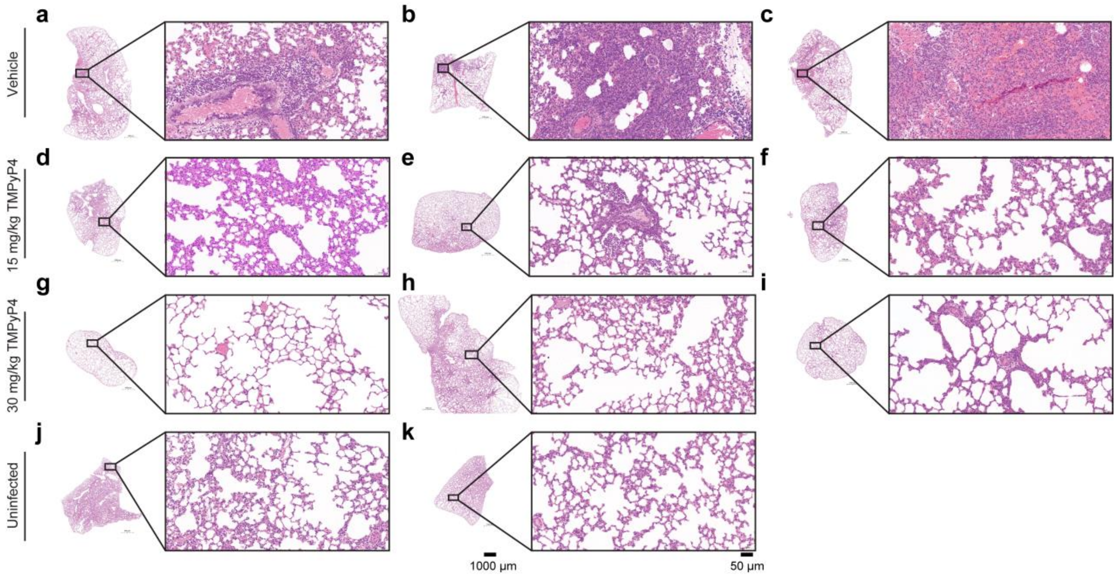
a
b
c
d
e
f
g
h
i
j
k
Vehicle
15 mg/kg TMPyP4
30 mg/kg TMPyP4
Uninfected
1000 μm
50 μm

Fig. S20 Scanning images of whole lung tissue sections. a-k The whole lung slide scan images from SARS-CoV-2 (10^5 $TCID_{50}$ virus/hamster)-infected (*n* = 3) or uninfected (*n* = 2) hamster on 3 days post infection. The corresponding higher-magnification images were shown. [Note: (c), (e), (g) and (k) correspond to the full images of the lung tissue sections of mice treated with vehicle, 15 mg/kg TMPyP4, 30 mg/kg TMPyP4 and uninfected shown in Fig. 6d, respectively].

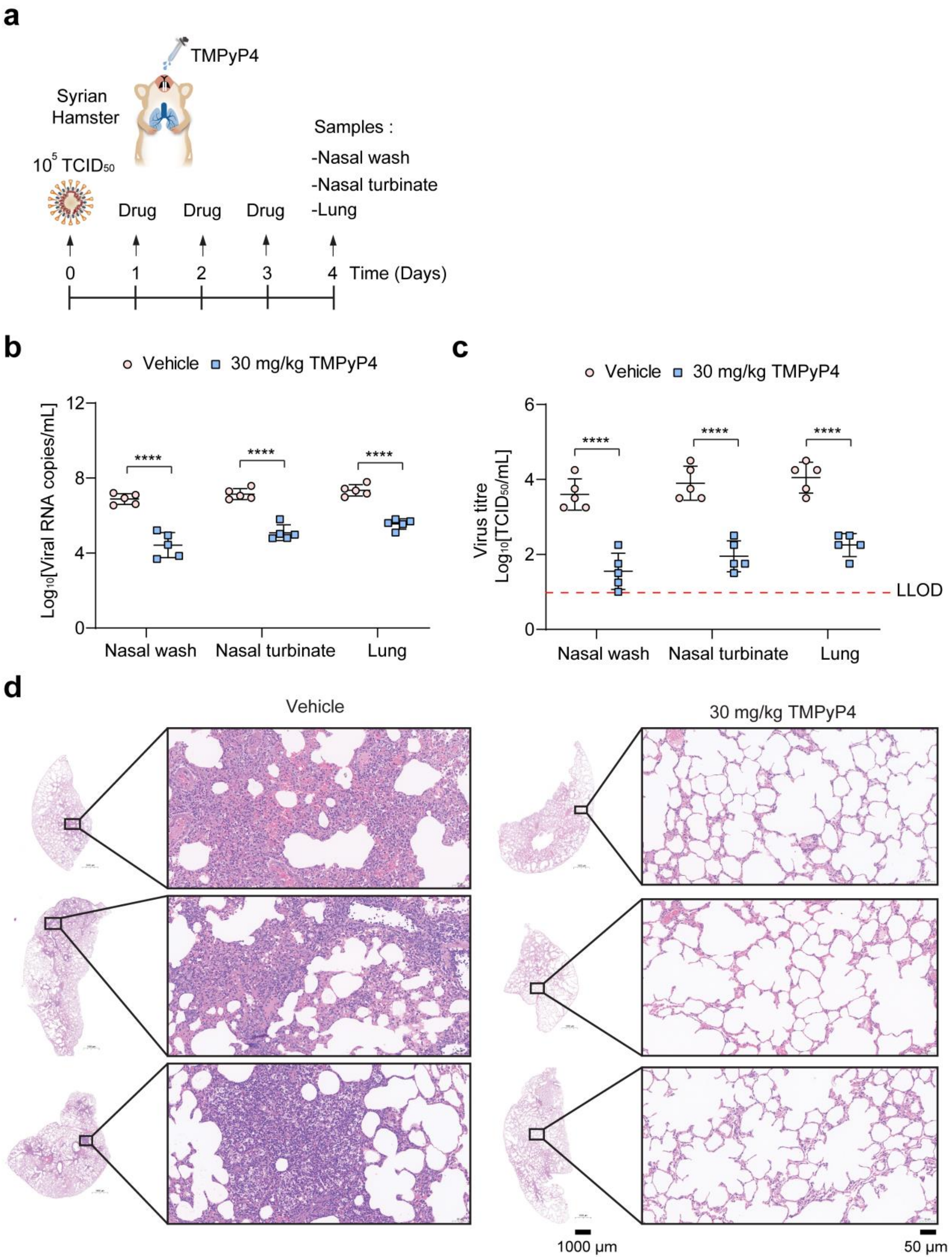
a
TMPyP4
Syrian Hamster
10^5 TCID50
Drug
Drug
Drug
Samples :
-Nasal wash
-Nasal turbinate
-Lung
0
1
2
3
4
Time (Days)
b
Vehicle
30 mg/kg TMPyP4
Log10[Viral RNA copies/mL]
12
8
4
0
****
Nasal wash
Nasal turbinate
Lung
c
Vehicle
30 mg/kg TMPyP4
Virus titre Log10[TCID50/mL]
6
4
2
0
****
LLOD
Nasal wash
Nasal turbinate
Lung
d
Vehicle
30 mg/kg TMPyP4
1000 μm
50 μm

Fig. S21 Antiviral activity of TMPyP4 in the hamster pre-infected with SARS-CoV-2. a Schematic of SARS-CoV-2 infected hamster model. Hamsters infected with SARS-CoV-2 (10^5 $TCID_{50}$ virus/hamster) were treated with vehicle (n = 5) or 30 mg/kg (n = 5) TMPyP4 for consecutive 3 days, with the first dose given at 24 h after infection with SARS-CoV-2. **b,c** Viral RNA copies (b) and viral titers (c) in the hamster nasal wash, nasal turbinate and lung tissues of the TMPyP4-treated groups relative to vehicle controls, determined by qRT-PCR and $TCID_{50}$ at day 3 after infection. LLOD for viral titers is indicated with a red dotted line. **d** The whole lung slide scan images from SARS-CoV-2 (10^5 $TCID_{50}$ virus/hamster) pre-infected hamster (n = 3) on 4 days post infection. The corresponding higher-magnification images were shown. Data are shown as mean ± SEM, two-tailed Student's t test. $^{****}P < 0.0001$.

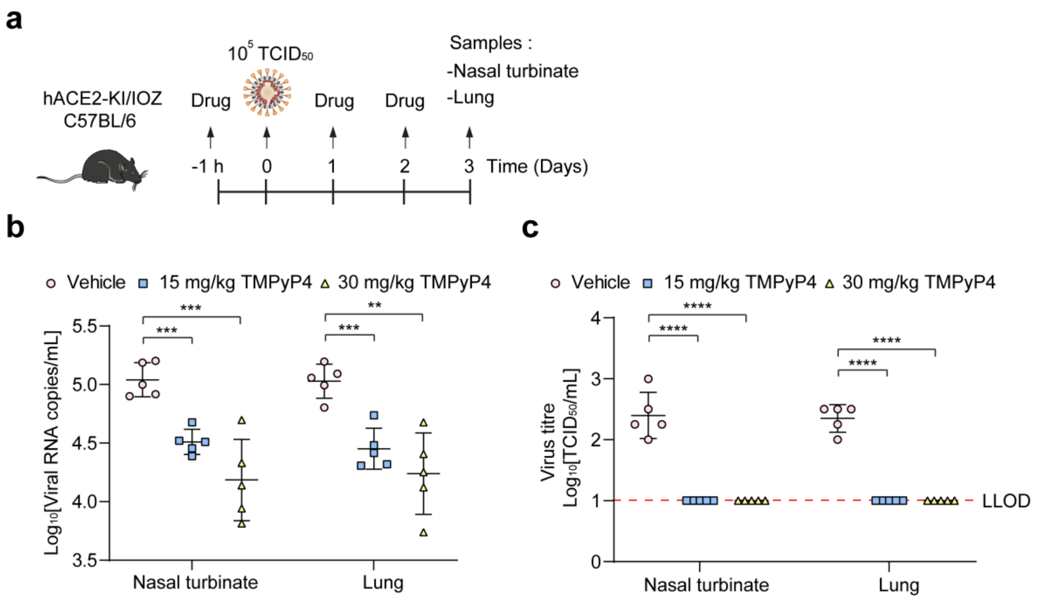
a
hACE2-KI/IOZ
C57BL/6
Drug
10^5 TCID50
Drug
Drug
Samples :
-Nasal turbinate
-Lung
-1 h
0
1
2
3
Time (Days)
b
Vehicle
15 mg/kg TMPyP4
30 mg/kg TMPyP4
Log10[Viral RNA copies/mL]
***
***
**
***
Nasal turbinate
Lung
c
Vehicle
15 mg/kg TMPyP4
30 mg/kg TMPyP4
Virus titre
Log10[TCID50/mL]
****
****
****
****
LLOD
Nasal turbinate
Lung

Fig. S22 Antiviral activity of TMPyP4 in hACE2 transgenic mouse model of SARS-CoV-2 infection. a Schematic of hACE2 transgenic mouse model of SARS-CoV-2 infection. Mice infected with SARS-CoV-2 (10^5 TCID50 virus/hamster) were treated with vehicle (n = 5), 15 mg/kg (n = 5) or 30 mg/kg (n = 5) TMPyP4 for consecutive 3 days, with the first dose given at 1 h before infection with SARS-CoV-2. **b,c** Viral RNA copies (b) and viral titers (c) in the mice nasal turbinate and lung tissues of the TMPyP4-treated groups relative to vehicle controls, determined by qRT-PCR and $TCID_{50}$ at day 3 after infection. Lower limit of detection for viral titers is indicated with a red dotted line. Data are shown as mean ± SEM, two-tailed Student's t test. $^{**}P < 0.01$, $^{***}P < 0.001$, $^{****}P < 0.0001$.

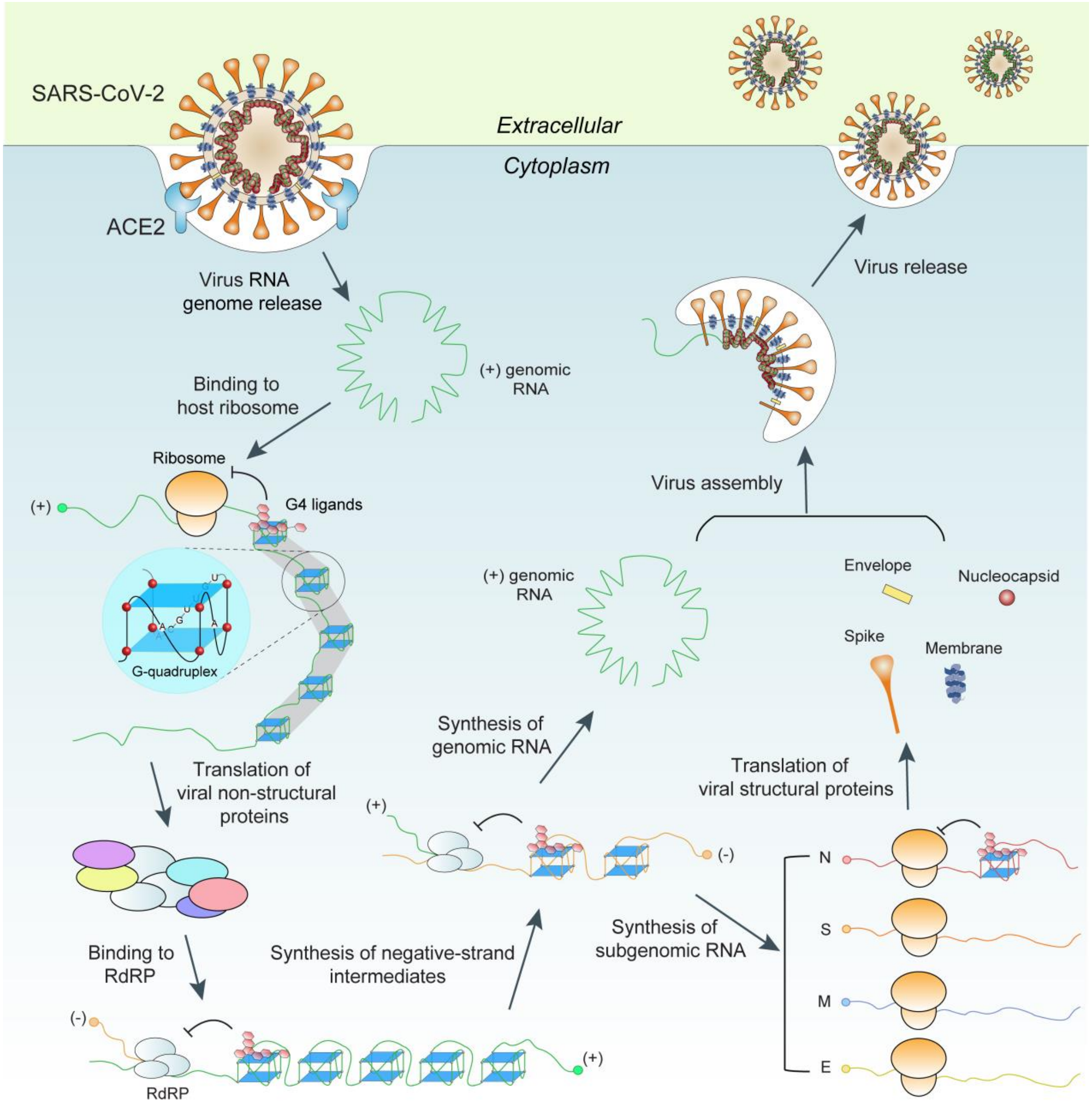
SARS-CoV-2
Extracellular
Cytoplasm
ACE2
Virus RNA genome release
(+) genomic RNA
Binding to host ribosome
Ribosome
G4 ligands
(+)
G-quadruplex
Translation of viral non-structural proteins
Binding to RdRP
(-)
RdRP
(+)
Synthesis of negative-strand intermediates
Synthesis of genomic RNA
(+)
(-)
(+) genomic RNA
Virus assembly
Virus release
Envelope
Nucleocapsid
Spike
Membrane
Translation of viral structural proteins
Synthesis of subgenomic RNA
N
S
M
E

Fig. S23 The regulatory mechanism of G4s in SARS-CoV-2 life cycle. G4s act as inhibition elements in the SARS-CoV-2 life cycle and inhibit both the viral replication and translation processes by impairing the elongating of RdRp and ribosomes, leading to hinder the production of viral RNA and proteins. G4-specific ligands can bind and stabilize the G4s to enhance the inhibitory effects, which is a promising antiviral therapeutic strategy for COVID-19.
